# An Overlooked Habitat‐Dependent Link Between Metabolism and Water Loss in Reptiles

**DOI:** 10.1111/1749-4877.13016

**Published:** 2025-07-21

**Authors:** Shahar Dubiner, Shai Meiri, Eran Levin

**Affiliations:** ^1^ School of Zoology, Faculty of Life Sciences Tel Aviv University Tel Aviv Israel; ^2^ The Steinhardt Museum of Natural History Tel Aviv University Tel Aviv Israel

**Keywords:** arid, humidity, hydroregulation, lizard, respirometry, snake

## Abstract

Maintaining the body's water balance is crucial for function and survival in all animals. Humidity conditions vary between different habitats and greatly affect an animal's evaporative water loss (EWL). Species inhabiting arid regions have adaptions to minimize water loss, which those adapted to life in humid regions may lack. Therefore, the physiology of species from different habitats could respond differentially to acute exposure to dry conditions. We measured the EWL and resting metabolic rates (RMRs) of 12 Israeli squamate species, from either mesic or xeric habitats, spanning four orders of magnitude in size. We treated the animals to dry and humid air simulating natural conditions (vapor pressure deficits 3 and 1 kPa, respectively) at an ecologically relevant temperature of 25°C. EWL rates were higher in dry air, and the effect was stronger in mesic species. EWL of mesic species in humid air is similar to EWL of xeric species in dry air, indicating similar EWL when tested under settings that match each species’ natural conditions. In dry air, the RMR of small‐bodied (<5 g) mesic species increased, whereas those of some small‐bodied xeric species decreased. Small mesic species might be displaying stress from unnaturally dry conditions, whereas small xeric species possibly display an adaptation to minimize EWL by lowering RMR, thereby respiration rates. Physiological measurements are usually taken in dry air, and our results suggest previous experiments may contain a methodological bias. Future ecophysiological research needs to consider ambient humidity, by either varying experimental humidity to match natural conditions, or considering possible effects of humidity during analysis and interpretation of experiments and models.

## Graphical abstract

We measured the gas exchange of six lizard and six snake species, under high and low air humidity, and two intriguing patterns emerged. First, although desert species lose less water via evaporation than similar‐sized mesic species under similar conditions, water loss is virtually the same when each is exposed to its natural conditions. Second, small‐bodied mesic species’ metabolic rates were higher in dry air (which could indicate stress from the unnatural conditions) but those of desert species were lower (possibly, an adaptation reducing respiratory water loss).

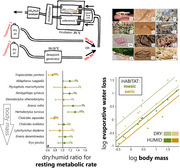

## 1 Introduction

Maintaining the body's water balance within tolerable limits is crucial for function and survival in all animals. Therefore, over evolutionary time scales, low water availability in the environment can drive adaptations to restrict water loss (Cox and Cox 2015). Both drinking water availability and air humidity strongly affect water balance. Dry air has a high water vapor pressure deficit (VPD), which increases an animal's water loss rate (Bulova 2002; Le Galliard et al. 2021; Mautz 1982) by physically driving higher evaporation from the body surface (Campbell and Norman 1998; Withers et al. 2016). However, direct links between air humidity and physiological parameters other than water loss, such as metabolic rate, are rarely studied (especially in ectotherms). Instead, they are implicitly treated as separate aspects of physiology that operate independently from water loss. For example, the vast majority of respirometric experiments are conducted in dry air for practical reasons (Lighton 2018), without considering it as an influencing factor. Furthermore, the most commonly used biophysical models (e.g., NicheMapR; Kearney and Porter 2009) do not typically consider humidity as having a direct effect on ectotherm energetics.

Humidity influences many aspects of physiology beyond water loss. For example, high relative humidity (RH) at a given temperature entails lower VPD and thus reduces the capacity for evaporative heat loss. In endotherms, this can have an effect on metabolic rates via changes to the required metabolic heat production. This effect was observed in some marsupials and birds (Cooper et al. 2020; Cooper and Withers 2008; Gilson et al. 2021; van Dyk et al. 2019) but not in others (Cooper et al. 2020; Gerson et al. 2014; Withers et al. 2022). Other aspects of physiology can respond to air humidity; for example, birds migrating in dry conditions can avoid dehydration by switching from fat to protein catabolism, which releases bound water (Gerson and Guglielmo 2011). Some amphibians reduce activity in dry air (Preest and Pough 1989) and select lower body temperatures (Galindo‐Martínez et al. 2018; Malvin and Wood 1991), to behaviorally reduce the VPD they experience (temperature is an important modulator of both water loss and metabolism in ectotherms). In reptiles, biophysical models show that dehydration (exacerbated by increased water loss in dry air) leads to dietary intake above what is energetically optimal in an attempt to regain water balance (Kearney et al. 2013), although dietary water was experimentally shown to be outweighed by the hydric cost of digestion (Chabaud et al. 2023; Lillywhite 2017; Murphy and DeNardo 2019; Wright et al. 2013). Some desert squamates exposed to high temperatures show lower metabolic rates than predicted, suggesting a mechanism that minimizes energy expenditure through respiratory cooling (Giacometti et al. 2022).

Compared to other land vertebrates, reptiles are less dependent on constant water availability and consequently are highly diverse in extreme deserts compared to other vertebrate groups (Raz et al. 2024; Roll et al. 2017). A combination of factors enables reptiles to maintain minimal levels of water loss. Their skin is dry, mostly glandless, and contains water‐conserving scales and lipid layers that reduce their cutaneous evaporative water loss (EWL; Lillywhite 2005; Mautz 1982; Roberts and Lillywhite 1980). In smaller animals, however, these adaptations can be outweighed by physical constraints on evaporation. For a small body, the surface area is greater relative to volume, which means a greater portion of the body is exposed to evaporation, leading to higher mass‐specific cutaneous EWL (Alexander 1996; Mautz 1982). Their mass‐specific metabolic rates are also higher, leading to higher respiration rates (Alexander 1996) which, in turn, increase the respiratory EWL (Loughran and Wolf 2023). Reptile EWL rates are also correlated with the water availability of their natural habitats (Bentley and Schmidt‐Nielsen 1966; Cox and Cox 2015; Dmi'el 2001; Gans et al. 1968; Mautz 1982). Although EWL is known to be plastic (Kattan and Lillywhite 1989; Sannolo et al. 2020; Weaver et al. 2023), mesic‐adapted species are generally less able to cope with xeric environments than species from more arid regions (Cox and Cox 2015).

Small‐bodied and mesic‐adapted reptiles are thus at risk of dehydrating in dry environments (e.g., at the edges of their distribution, during droughts, or due to changes in climate and habitat structure), and these conditions could potentially act as a stressor. Since stress in reptiles is often accompanied by an increase in metabolic activity (DuRant et al. 2008; Meylan et al. 2010; Richard 2020), measurements of resting metabolic rates (RMRs) conducted under stressful conditions would thus not reflect true “resting” rate as assumed by the researchers. Other physiological responses to dryness could elevate their metabolic rates as well, for example, catabolizing protein to counteract dehydration (Brusch et al. 2018; Dezetter et al. 2021; Dubiner et al. 2024a; Gerson and Guglielmo 2011; but see de Souza et al. 2004). We aimed to elucidate the effects of experimentally controlled humidity, biogeographic humidity (i.e. whether species inhabit mesic or regions) body size, and their interactions, on squamate water loss and energetic expenditure. Specifically, we hypothesized that RMR would not differ between habitats but would be higher in response to dry conditions in species with higher risk from water loss (small and mesic‐adapted species), for which these conditions are stressful. We also hypothesized that the EWL of mesic species will be higher (Cox and Cox 2015) and will be more affected by exposure to dry conditions.

## 2 Materials and Methods

### 2.1 Study Species

We studied the water loss and metabolic rates of three gecko species, three skink species, three fossorial snake species (two scolecophidians and one boid), and three colubrid species (Appendix 1) in the lab. We used common and non‐threatened reptile species in our experiments. These species were chosen to span four orders of magnitude in body size and two distinct biomes. The first is the mesic Mediterranean biome, which, in Israel, is characterized by relatively cool rainy winters, with 250–800 mm rainfall, and hot dry summers. The second is the xeric desert biome, which is dry throughout most of the year, with hot days and rainfall events limited to a few events in winter and totaling 20–200 mm annually (Israel Meteorological Service: https://ims.gov.il/en).

Xeric (desert) adapted species in our study were: (1) The nocturnal gecko *Tropiocolotes yomtovi*, Israel's smallest reptile (mean mass in our sample was 0.37 g), inhabits deserts in southern Israel and eastern Sinai (Ribeiro‐Júnior et al. 2022; Bar et al. 2021). (2) *Stenodactylus sthenodactylus* (2.3 g) is a nocturnal gecko common in the Sahara Desert and Southern Israel (Bar et al. 2021). (3) The nocturnal psammophilic skink *Chalcides sepsoides* (4.7 g) is distributed across the northeastern Saharan desert and, in Israel, also along the Mediterranean coastal dunes (Bar et al. 2021). (4) *Myriopholis macrorhyncha*, Israel's smallest snake (0.6 g), is a nocturnal and fossorial thread snake. It is likely a species complex, with various lineages inhabiting deserts from the Sahara to India (Bar et al. 2021; Roberta Graboski, personal communication). (5) The colubrid *Lytorhynchus diadema* (18.4 g) is nocturnal and psammophilic, distributed in the Saharo‐Arabian deserts including southern Israel. In Israel, it extends northward along the Mediterranean coastal dunes (Bar et al. 2021).

Mesic (Mediterranean) adapted species in our study were: (6) *Xerotyphlops syriacus*, a small (mean mass in our sample was 1.9 g) nocturnal blind snake common across the Levant (Bar et al. 2021). (7) *Eryx jaculus* (65.5 g) is a semi‐fossorial, mostly nocturnal boid, inhabiting mesic habitats across Southeastern Europe and North Africa (Bar et al. 2021). In Israel, it inhabits the mesic Mediterranean region. (8) *Hemidactylus turcicus* (2.9 g) is a circum‐Mediterranean nocturnal gecko. In Israel desert and Mediterranean populations form two distinct clades (Karin Tamar, pers. comm.). Our experiments were solely conducted on individuals from the northern, mesic clade to avoid introducing known physiological differences between populations of this species (Vardi et al. 2023). (9) The mostly diurnal skink *Chalcides ocellatus* (10.5 g) likewise inhabits all of Israel—but all individuals we experimented on come from mesic Mediterranean populations to avoid known population differences (Reut Vardi, unpublished). (10) *Ablepharus rueppellii* (0.51 g) is a diurnal skink mostly inhabiting mesic regions in Israel (Bar et al. 2021; all our individuals came from mesic parts of its distribution). (11) *Eirenis rothii* (2.7 g) and (12) *Eirenis decemlineatus* (29.9 g) are cathemeral and diurnal colubrids, respectively, inhabiting mesic Mediterranean regions in the Levant (Bar et al. 2021). All lizard species and *L. diadema* are active from spring to autumn. All other snakes are predominantly active in the spring (personal observations). See Appendix 1 for images and phylogenetic tree.

### 2.2 Animal Collection and Field Measurements

Animals were collected under permits #42875, #43032, and #43553 from the Israel Nature and Parks Authority. We aimed to obtain six individuals for each species, but only five individuals of some snake species were caught. For collection sites, see Table S1. We kept the animals in terraria at around 26°C, with substrate taken from their collection site, and provided them with water *ad libitum* for 2 to 3 days before each experiment and the night between the two replications. At the end of the experiments, the animals were given food and water *ad libitum* for one night and released the following evening back to the location they were captured in.

**FIGURE 1:**
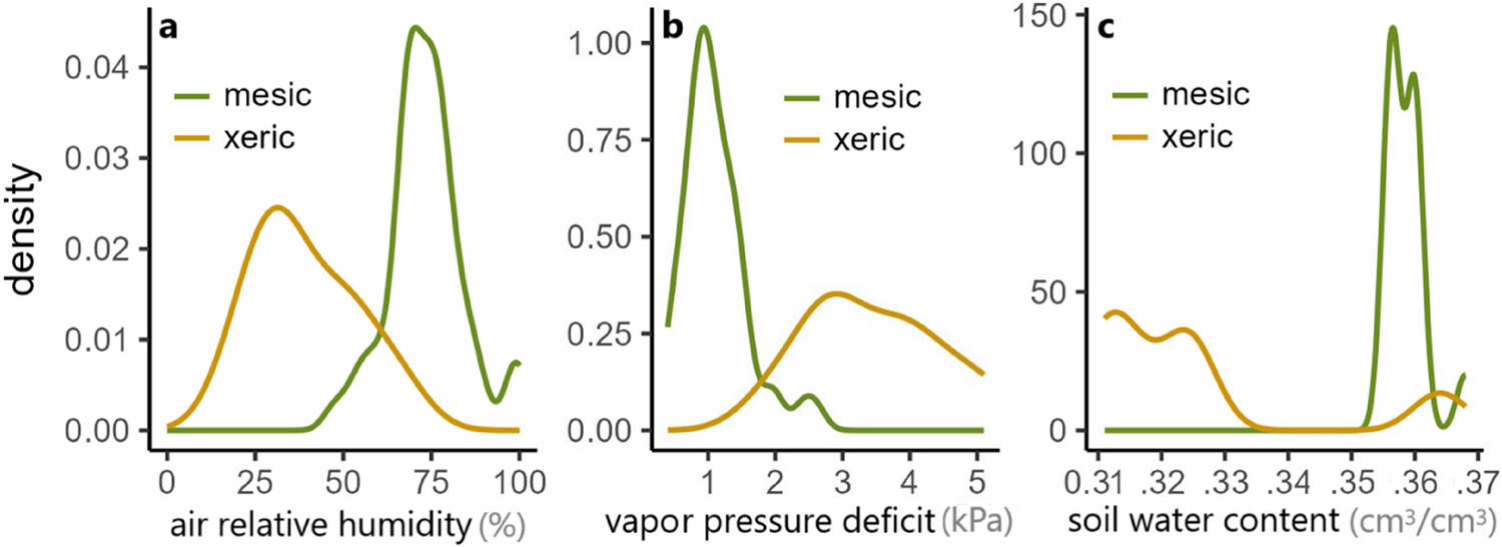
Smoothed histograms (density plots) of (a) air relative humidity and (b) vapor pressure deficit under shelters where individuals from our study species were found during field excursions. All seven mesic species were closely distributed around 70% RH = 1 kPa VPD, leading us to choose these as our “humid” treatment. Xeric species are only represented in plot (a) by *Tropiocolotes yomtovi* and *Chalcides sepsoides*, since the other xeric species were rarely located inactive under shelters in the field; however, the other desert species inhabit similar sites. (c) soil water content at 0–5 cm depth (−10 kPa, obtained from www.soilgrids.org) at the coordinates where we collected each of the individuals for the experiment. See Table S2 for underlying data.

To determine differences in the natural humidity conditions experienced by mesic and arid‐adapted species, we measured relative humidity (RH, Figure 1a) and temperature (to convert to VPD, Figure 1b) under shelters where our study individuals were found in nature. Resting animals were located by flipping rocks. When an animal was caught, we moved it to a temporary shelter and immediately took the substrate temperature at the spot where the animal had been, using a shaded digital thermometer (Kamtop, USA; accuracy within 1.5%). We then proceeded to measure the relative humidity using an IBS‐TH2 Plus humidity sensor (±3% accuracy; Inkbird Tech, China) by placing the sensor at the spot where the animal had been spotted, covering it with a plastic cap (diameter 10 cm, height 2 cm) and replacing the rock. We waited for 5–10 min for the RH reading to stabilize before recording it. Microhabitat conditions were gathered, independently of animal collection, between the spring of 2022 and the summer of 2024, during the seasons in which each species is active. Additionally, we obtained the water content in the soil (cm^3^/cm^3^ at 0–5 cm depth, −10 kPa; Figure 1c) from a global map (www.soilgrids.org), at the coordinates where we collected animals for the experiment (0.25 km grid). See Table S2 for coordinates and full data. We stress that the characteristic values of natural air and soil humidity were not directly used in the experiments and analysis. These served only to support our classification of species and populations as arid or mesic‐adapted and to set the relative humidity conditions in the respirometric trials so they reflect the common natural conditions of their habitats.

### 2.3 Respirometric Trials

We report our respirometric and statistical methods based on the guidelines recommended by Wu et al. (2024) for openness and transparency of metabolic rate and evaporative water loss data. Each individual was measured separately in a plastic metabolic chamber connected to a constant CO_2_‐free airflow, kept at 25°C (an ecologically relevant temperature for all of these species; Dubiner et al. 2024b and personal observation) using a Peltier incubator (Thermo Scientific, USA). Incoming air was set to 50 mL/min, and one of two treatments: dry air (RH < 10%, supplied from a cylinder and passing through a column of silica gel), and humid air at 70% RH achieved by passing the dry air through an LI‐610 portable dew point generator (LICOR, USA) set to 19.15°C (calculated to give the above RH). These conditions are equivalent to a vapor pressure deficit of 0.95 kPa in the humid and ∼3 kPa in the dry treatment, approximating the conditions commonly found in nature for our mesic species at rest (Table S2). The Chamber size was 750 mL for the larger species (*C. ocellatus*, *E. jaculus*, *L. diadema*, and *E. decemlineatus*) and 50 mL for all other species. Flow rates were 200 or 50 mL/min, respectively. Rates were low to not perturb the animals, yet high enough to enable adequate turnover of the chamber air at the timescale of the experiment (98% turnover times of <4 min for the small chamber and <15 min for the large one; see eq. 8.1 in Lighton, 2018). Flow rates were continuously verified at the output using an ALIM‐2000CCM flow meter (Alicat, USA). Each animal was measured twice in each treatment so that each of the two treatments was given on the same day but the two replicates were conducted on different days. Animals were measured one at a time. To avoid bias due to treatment order, for example, by residual water carried over in the skin from one trial to the next, individuals were divided into two randomized blocks. Half of the individuals received the humid treatment followed by dry in the first replicate, and the dry treatment followed by humid in the second replicate, and the other individuals received the treatments in the opposite order.

Air exiting the chamber flowed into an LI‐7000 H_2_O/CO_2_ analyzer (LICOR, USA), from which readings were recorded using a UI‐3 data acquisition interface and Expedata version 1.9.20 (Sable Systems, USA). The baseline for V̇H_2_O and V̇CO_2_ values were obtained from an empty but otherwise identical reference chamber, which received the same airflow concurrently with the measurement and was recorded for 10 min between every 30 min of measurement (the switch between them was done automatically using an RM‐8 multiplexer). We calculated evaporative water loss (EWL, units of mg H_2_O/hour) from mean V̇H_2_O values during 30 min of resting, corrected according to the baseline V̇H_2_O values and an empirically generated calibration curve. The calibration curve was created using the methods of Dubiner et al. (2024c), by measuring the evaporation of known quantities of water (1–20 µL) injected directly into the metabolic chamber and regressing these values against the observed values (repeated twice, *R* ^2^> 0.99). We obtained RMRs (units of µL O_2_/hour) during 30 min of resting, calculated from mean V̇CO_2_ values according to eq. 10.2 in Lighton (2008). Values of V̇CO_2_ are adjusted for H_2_O in the LI‐7000, and we corrected them using eq. 11.4 in Lighton (2008) according to the baseline CO_2_ and assuming a reasonable RQ (respiratory quotient) value of 0.8 (Koteja 1996). V̇CO_2_ rather than V̇O_2_ was used for determining RMR because O_2_ measurements are more sensitive to water in the air (Lighton 2008) and for better precision (especially in the small species where changes in O_2_ are hard to detect; Lighton and Halsey 2011). Furthermore, the LI‐7000 corrects CO_2_ for water vapor content, which is measured concurrently in the same device. For these reasons, even if it sacrifices overall accuracy by a few percent, basing our estimate of RMR on CO_2_ is much less likely to introduce bias with respect to air humidity, which is the focus of this study. However, to ensure that any differences in RMR between treatments are not an artifact of the CO_2_ measurement method, throughout the experiments for two species (*E. rothii* and *X. syriacus*), we passed the exiting air from the LI‐7000 through a magnesium perchlorate‐Ascarite column (water and CO_2_ scrubbers) and into an Oxzilla O_2_ analyzer (Sable Systems, USA). This served as a validation that we observed similar trends using different ways of obtaining RMR. See Appendix 2 for the experimental setup diagram.

We validated both treatments periodically, by repeating measurements with no animal in the measurement chamber, to ensure that the results were not skewed by different readings between the chambers or channels. For a few measurements, we validated that air from the tank was dry (RH <10%) by placing the IBS‐TH2 Plus humidity sensor in the metabolic chamber at 200 mL/min airflow. We validated that the temperatures were indeed 25°C by measuring both the airflow and animals’ body temperatures following a random subset of the experiments using a thermocouple (TC‐2000, Sable Systems USA). We assumed a resting state when the V̇CO_2_ and V̇H_2_O throughout the measurement were stable and the animal was completely motionless for its duration (surveilled by an infrared camera, for the entire duration of the experiment), a state normally achieved within the first hour. We waited for 30 full minutes of resting state to elapse, and then used the following 30 min for our analyses; therefore, the experiment lasted about 2 to 2.5 h per animal. In the rare cases, the resting state was not achieved after 90 min in the chamber, the experiment was terminated and its results were discarded. All measurements were conducted under ethics permit #18616 from the TAU Ethics Committee.

### 2.4 Statistical Analysis

For each species, we ran generalized linear mixed models (GLMMs, gamma distribution with a log link function), one for EWL and another for RMR, with body mass (g) and treatment (dry or humid) as predictors. Random factors were individual (ID), treatment order (dry then humid, or vice versa), block (group of animals from the same site, measured on the same day in random order), and replicate (first vs. second repetition of the experiment). In two cases where an animal defecated, one case when it had recently lost its tail, or there were technical errors in V̇H_2_O recording (six cases, all in the humid treatment), but the RMR reading was valid, the EWL of the specific measurement was excluded from the analysis but the RMR value was still used.

We ran phylogenetic generalized linear mixed models (PGLMMs, using the R package “phyr”; Li et al. 2020) of all species together, one with mean species EWL and another for mean species RMR (both log‐transformed) as the response variables. We included interactions of species body mass (g, log‐corrected, average for the individuals tested), treatment (dry or humid), and natural habitat (mesic or xeric) as predictors. We accounted for phylogenetic non‐independence using the time‐calibrated phylogenetic tree (Appendix 1) from Zheng and Weins (2016). As a sensitivity analysis, we re‐ran the models with all individual measurements rather than species means (with species, individual, treatment order, block, and replicate as random factors). All models were tested for fit and residual autocorrelation. *R* ^2^ for the mixed models was estimated by maximum likelihood using the “R2_lik” function in the R package “rr2” (Ives and Li 2018). See File S1 for the R code.

## 3 Results

### 3.1 Species‐Specific Models

**FIGURE 2:**
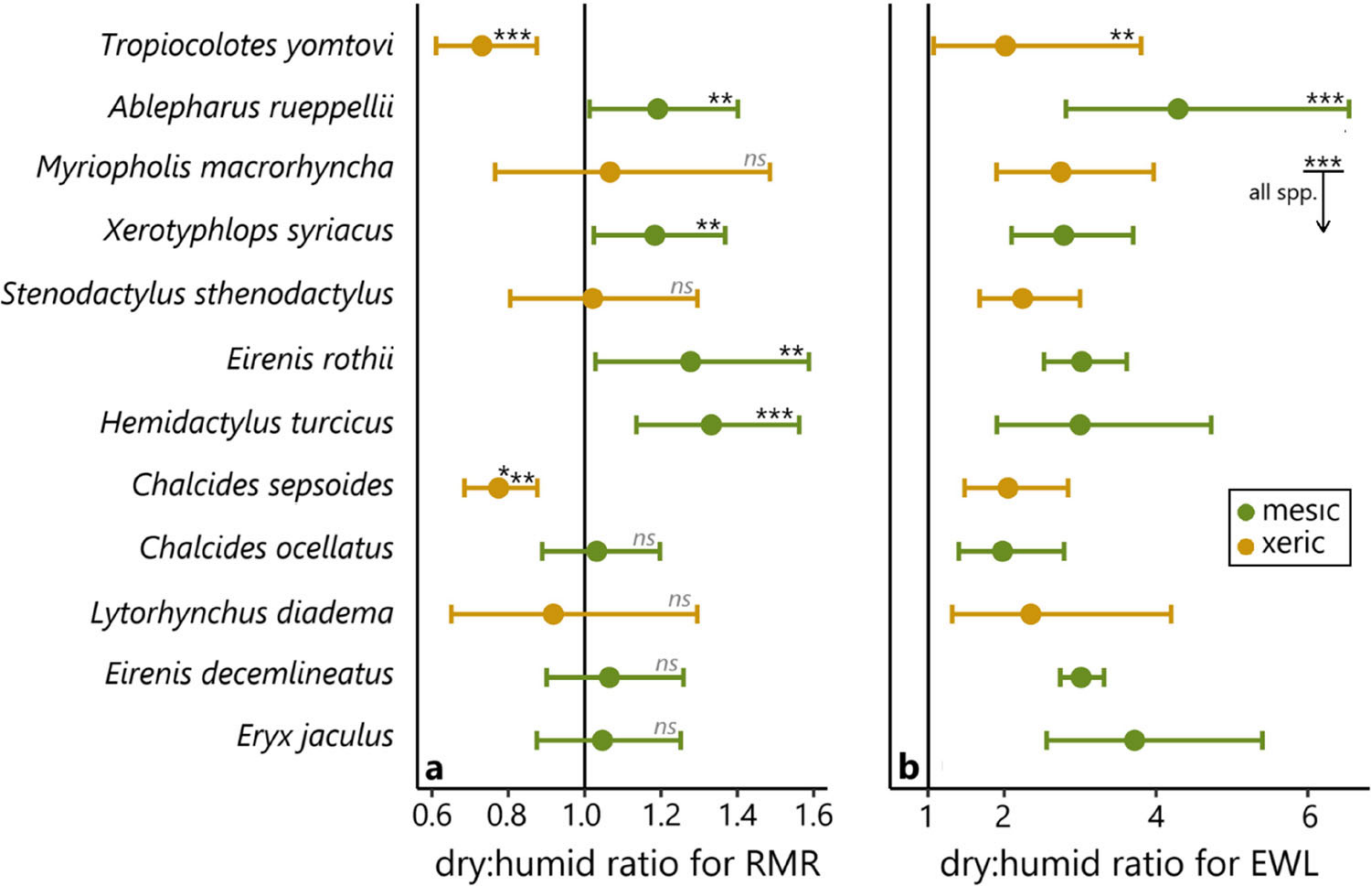
Mean (points) and 95% CI (error bars) of resting metabolic rate (a) and evaporative water loss (b) in the dry air treatment compared to the humid treatment (i.e., dry:humid ratio for each result), according to the GLMMs (*n* = 5–6 individuals per species); **: *p* < 0.01; ***: *p* < 0.001; *ns*: not significant. Species are ordered top‐down from smallest to largest mean body mass.

Mass‐specific RMR (expressed as O_2_ consumption rate per unit mass) in the dry treatment ranged from 20.4 ± 6.8 µL/g/h in the mesic snake *E. jaculus* to 126.7 ± 30.3 µL/g/h in the small mesic skink *A. rueppellii*. Specific RMR in the humid treatment ranged from 19.7 ± 10.0 µL/g/h (mean ± SD) in *E. jaculus* to 129.3 ± 33.9 µL/g/h in the tiny xeric gecko *T. yomtovi*. See Table 1a and Table S3 and Appendix 3 for complete results, and Table S1 for the raw data. In dry air, RMR increased significantly for the four smallest mesic species, *A. rueppellii*, *X. syriacus*, *E. rothii*, and *H. turcicus*. RMR decreased significantly in dry air for two of the xeric species, *T. yomtovi*, and *C. sepsoides* (GLMM: *p* < 0.01 for all six species; Figure 2a). Validation of these results for two species (*E. rothii* and *X. syriacus*), using O_2_ rather than CO_2_ to calculate RMR, yielded very similar results (Table S4). RMR did not significantly change with air humidity for the other six species. Note that, contrary to the positive effect of dry air on mesic species, its effect on xeric species is not unequivocally a trait of extremely small species: the 4.7 g *C. sepsoides* exhibited significant differences, whereas the 0.6 g *M. macrorhyncha* did not.

**TABLE 1:** Results of the measurements for all species, resting metabolic rate (RMR, a) and evaporative water loss (EWL, b). Results are mean ± SD, and “effect” is the generalized linear mixed model (GLMM) prediction for the difference between the dry and humid (70% relative humidity) treatments. Significant differences (*p* < 0.05) are shown in **bold**. Sample sizes (*n*) for EWL, in the three species where *n* differed between treatments due to technical issues (see Methods section), are presented as *n* for humid(dry). Note that some xeric species’ ranges extend north to sandy habitats along the coastal dunes. Species collected mesic habitats, but whose distributions in Israel extend across both the Mediterranean and desert biomes, are marked with an asterisk: “mesic*.”

(a) Resting metabolic rate	*n*	Family	Habitat	Mass (g)	RMR (µL O_2_/g/h)		GLMM		
Species					Dry air	70% RH	Effect	*t*	*p*
*Tropiocolotes yomtovi*	6	Gekkonidae	xeric	0.33 ± 0.06	96.0 ±28.4	129.3 ±33.9	−26.9%	**−4.26**	**<0.001**
*Stenodactylus sthenodactylus*	6	Gekkonidae	xeric	2.25 ± 0.87	92.8 ±31.5	94.6 ±38.1	+2.1%	0.22	0.829
*Hemidactylus turcicus*	6	Gekkonidae	mesic*	2.93 ± 0.30	96.9 ±14.9	74.1 ±13.7	+33.2%	**4.40**	**<0.001**
*Ablepharus rueppellii*	6	Scincidae	mesic	0.51 ± 0.08	126.7 ±30.3	103.8 ±23.7	+19.2%	**2.64**	**0.008**
*Chalcides sepsoides*	6	Scincidae	xeric	4.73 ± 0.50	32.6 ±5.85	42.2 ±4.00	−22.5%	**−5.08**	**<0.001**
*Chalcides ocellatus*	6	Scincidae	mesic*	10.45 ±2.70	32.4 ±12.8	31.6 ±17.0	+4.7%	0.72	0.475
*Myriopholis macrorhyncha*	5	Leptotyphlopidae	xeric	0.60 ± 0.17	61.9 ±19.4	58.0 ±15.2	+6.6%	0.50	0.618
*Xerotyphlops syriacus*	6	Typhlopidae	mesic	1.93 ± 0.51	35.3 ±5.24	30.1 ±6.10	+19.2%	**2.85**	**0.004**
*Eryx jaculus*	5	Boidae	mesic	65.54 ±23.7	20.4 ±6.83	19.7 ±10.0	+4.7%	0.53	0.514
*Eirenis rothii*	6	Colubridae	mesic	2.68 ± 0.06	36.3 ±6.44	28.5 ±5.45	+27.8%	**2.76**	**0.006**
*Eirenis decemlineatus*	5	Colubridae	mesic	29.90 ±13.6	43.3 ±21.4	41.5 ±21.8	+6.5%	0.97	0.335
*Lytorhynchus diadema*	5	Colubridae	xeric	18.42 ±4.58	30.5 ±8.52	33.2 ±11.5	−8.2%	−0.64	0.523

(b) Evaporative water loss					EWL (mg H_2_O/g/h)		GLMM		
Species	*n*	Family	Habitat	Mass (g)	Dry air	70% RH	Effect	*t*	*p*
*Tropiocolotes yomtovi*	6	Gekkonidae	xeric	0.33 ± 0.06	0.91 ± 0.50	0.48 ± 0.40	+101.6%	2.71	**0.007**
*Stenodactylus sthenodactylus*	6	Gekkonidae	xeric	2.25 ± 0.87	0.89 ± 0.06	0.41 ± 0.06	+124.2%	6.79	**<0.001**
*Hemidactylus turcicus*	6	Gekkonidae	mesic*	2.93 ± 0.30	1.24 ± 0.44	0.41 ± 0.22	+199.9%	5.91	**<0.001**
*Ablepharus rueppellii*	5(2)	Scincidae	mesic	0.51 ± 0.08	2.46 ± 1.83	0.46 ± 0.14	+329.1%	8.45	**<0.001**
*Chalcides sepsoides*	6	Scincidae	xeric	4.73 ± 0.50	0.21 ± 0.03	0.10 ± 0.02	+104.9%	5.37	**<0.001**
*Chalcides ocellatus*	5(6)	Scincidae	mesic*	10.45 ± 2.70	0.38 ± 0.16	0.21 ± 0.17	+97.7%	4.86	**<0.001**
*Myriopholis macrorhyncha*	5	Leptotyphlopidae	xeric	0.60 ± 0.17	0.94 ± 0.30	0.35 ± 0.10	+174.6%	7.04	**<0.001**
*Xerotyphlops syriacus*	6	Typhlopidae	mesic	1.93 ± 0.51	0.95 ± 0.04	0.35 ± 0.08	+178.4%	8.83	**<0.001**
*Eryx jaculus*	5	Boidae	mesic	65.54 ± 23.7	0.23 ± 0.03	0.08 ± 0.05	+271.8%	9.03	**<0.001**
*Eirenis rothii*	6	Colubridae	mesic	2.68 ± 0.06	1.22 ± 0.18	0.42 ± 0.11	+202.1%	>10	**<0.001**
*Eirenis decemlineatus*	5(3)	Colubridae	mesic	29.90 ± 13.6	0.53 ± 0.20	0.15 ± 0.05	+201.3%	>10	**<0.001**
*Lytorhynchus diadema*	5	Colubridae	xeric	18.42 ± 4.58	0.21 ± 0.12	0.08 ± 0.03	+135.0%	3.79	**<0.001**

The lowest mass‐specific EWL (expressed as H_2_O evaporation rate per unit mass) was measured in the xeric colubrid snake *L. diadema*, with 0.08 ± 0.03 mg/g/h (mean ± SD) in the humid treatment and 0.21 ± 0.12 mg/g/h in the dry treatment. The highest specific EWL was measured in the small mesic skink *A. rueppellii*, with 0.46 ± 0.14 mg/g/h in the humid treatment and 2.46 ± 1.83 mg/g/h in the dry treatment. See Table 1b, Table S3, and Appendix 3 for complete results, and Table S1 for the raw data. The total EWL of all 12 squamate species we tested was significantly higher in the dry treatment (GLMM: *p* = 0.007 for *T. yomtovi* and < 0.001 for all other species). The magnitude of difference ranged from 97.7% to 321.1% in the mesic skinks *C. ocellatus* and *A. rueppellii*, respectively (Figure 2b).

### 3.2 Phylogenetic Comparative Models

**FIGURE 3:**
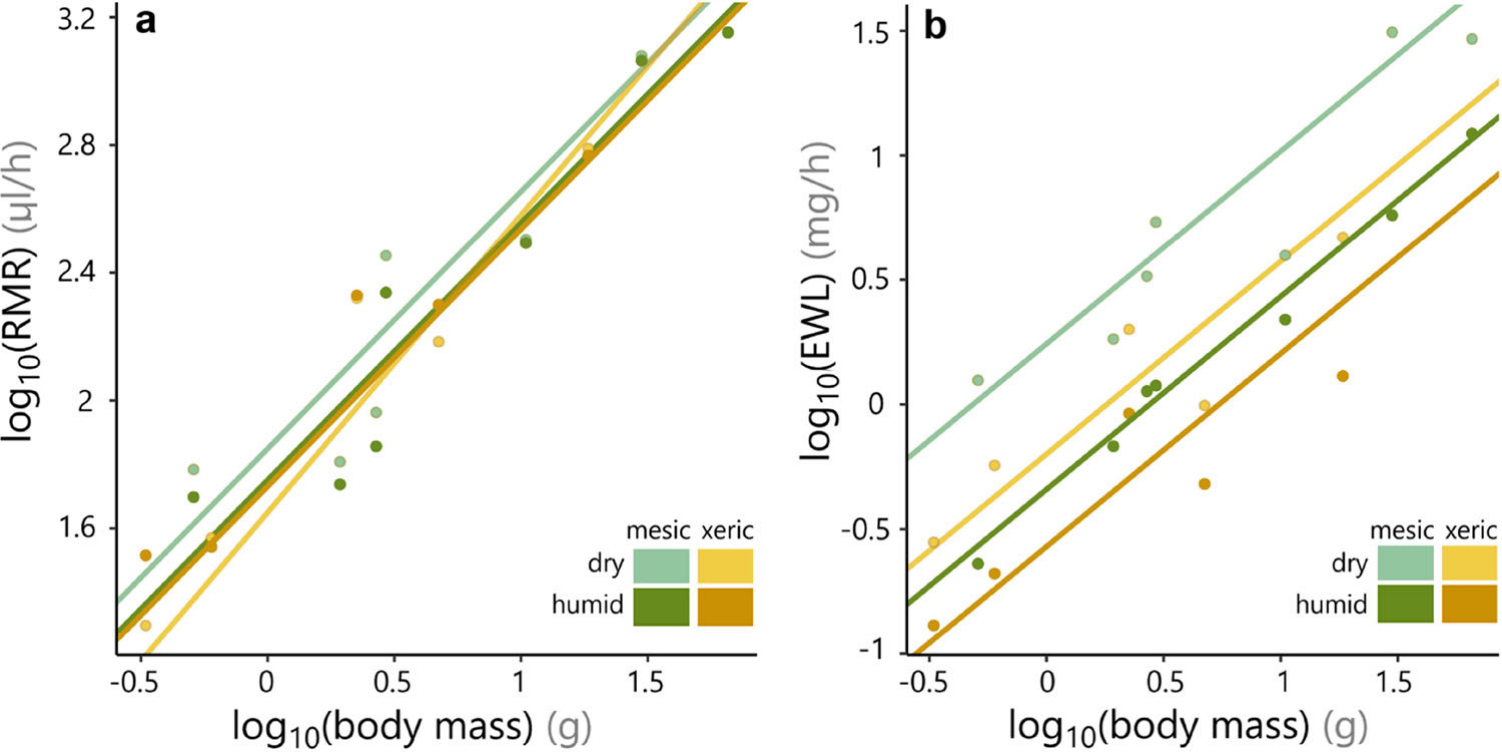
Species means and trendlines calculated from the PGLMMs (each point represents a species mean, *n* = 5–6 individuals). Resting metabolic rate (a) scales at an allometric exponent of 0.81. Differences in RMR between habitats are only significant in the dry treatment (*p* < 0.001). Evaporative water loss (b) scales with body mass at an exponent of 0.77. EWL is significantly higher in the dry treatment for both habitats (*p* < 0.001) and higher in the mesic habitat for both the dry (*p* < 0.001) and humid (*p* = 0.025) treatments. This makes the EWL for mesic species in humid (i.e., natural) conditions similar to that of xeric species in dry (i.e., natural) conditions.

According to the PGLMM for metabolic rate (*R* ^2^ = 0.97), the magnitude of the difference between RMR in dry versus humid air was influenced by the interaction between body size and habitat (*p* < 0.001; Table S5a). RMR increased with body mass at an allometric slope of 0.81 (±0.09; *p* < 0.001). For mesic species, RMR was 1.25 times higher in the dry treatment (*p* = 0.003; the effect is weakened by 0.12 per every tenfold increase in body mass, but not significantly: *p* = 0.093). In contrast, in the dry treatment, xeric species RMRs were lower (by 1.51 at the intercept of 1 g, *p* < 0.001) but scaled allometrically with a steeper slope (0.87, *p* = 0.017). Thus, RMR was lower for xeric compared to mesic species in the dry (*p* < 0.001), but not in the humid treatment (*p* = 0.457) nor for large‐bodied species (Figure 3a). The variance of random effects was 0.019 for species and 0.003 for their phylogenetic distances; the residual variance was 0.002 and there was no residual autocorrelation (Moran's *I* = −0.15; *p* = 0.599). The sensitivity analysis, which included all individual measurements rather than species means, showed qualitatively similar results, and the same significant trends for treatment (*p* = 0.011) and treatment–habitat interaction (*p* = 0.003; Table S5b).

The PGLMM for water loss (*R* ^2^ = 0.96; Table S5c) showed that EWL increased with body mass with an allometric exponent of 0.77 (±0.07; *p* < 0.001). This exponent did not differ significantly between habitats (*p* = 0.137) or treatments (dry vs. humid air; *p* = 0.272). EWL in humid air was, on average, 1.69 times higher for mesic compared to xeric species (controlling for body size; *p* = 0.025). EWL was higher in dry air in both environments (*p* < 0.001), but the magnitude of the difference was habitat‐dependent (interaction *p* = 0.036), increasing by a factor of 3.83 in mesic species but only by 2.35 in xeric species, widening the gap between habitats to 2.41‐fold in dry air (*p* < 0.001; Figure 3b). The variance of random effects was 0.002 for species and 0.007 for their phylogenetic distances; residual variance was 0.009 and there was no residual autocorrelation (Moran's *I* = −0.12; *p* = 0.765). The sensitivity analysis, which included all individual measurements rather than species means, showed qualitatively similar results, and the same significant trends for treatment (*p* < 0.001), habitat (*p* = 0.013), and treatment–habitat interaction (*p* = 0.041; Table S5d).

## 4 Discussion

RMRs scaled allometrically with body size, with an exponent of 0.81, similar to previous findings (Andrews and Pough 1985; Dubiner et al. 2023; Glazier 2005). Congruent with our hypothesis, the high EWL in dry air exhibited by the small‐bodied, mesic‐adapted species (*A. rueppellii*, *X. syriacus*, *E. rothii*, and *H. turcicus*) was accompanied by a significant increase in RMR (by 19%–33%). Such an increase was not observed in either larger or xeric‐adapted species. While RMR is not a standard metabolic rate (which measures the absolute minimal energy requirements), the animals were confirmed to be motionless, and therefore, this increase is not due to activity cycles or escape attempts. We hypothesize that mesic species that do not commonly encounter dry air in their microhabitat are experiencing the dry conditions as an environmental stressor. This could lead them to initiate a physiological stress response under dry conditions, which would elevate their metabolic rates (DuRant et al. 2008; Kepas et al. 2023; Meylan et al. 2010; Richard 2020). Although body size as a continuous predictor did not explain the magnitude of difference in RMR (interaction *p* = 0.093), all small mesic species (mass 0.5–2.9) experienced shifts, while none of the larger species did (mass 10.5–65.5 g). This can be explained by the higher susceptibility of small‐bodied (Alexander 1996; Mautz 1982) and mesic (Cox and Cox 2015; Mi et al. 2022) reptiles to desiccation, leading these species, but not others, to experience dryness as a stressor.

The xeric species did not have higher RMR in dry air. We hypothesize that, since these are their natural conditions, they should not be stressful to them. Remarkably, and not predicted by our hypothesis, the small, xeric gecko (*Tropiocolotes yomtovi)* and skink (*Chalcides sepsoides)* did have significant differences in RMR between dry and humid air, but the effect was in the opposite direction to the mesic species. RMRs were lower in dry air for these species (by 23%–27%) compared to their RMRs in humid air. Higher RMR in humid air for xeric species may represent a stress response to the unfamiliar humid conditions. However, we suggest that the lower RMR in dry air for these species is an adaptive mechanism for maintaining low EWL (as described above, the increase in EWL was weaker in xeric species). Small (or extremely small, e.g., the 0.3 g *T. yomtovi*) squamates in hot dry habitats are naturally exposed to extreme dehydration risk. Common adaptations to aridity (such as higher epidermal lipid content; Roberts and Lillywhite 1983) might be insufficient because temperatures in their resting sited can be extremely high. In the dry desert habitat, this will greatly increase the water vapor pressure deficit and elevate their EWL (Campbell and Norman 1998; Withers et al. 2016). By reducing their metabolic rate when resting in dry conditions, these species appear to reduce their respiration rates, thereby keeping water loss at manageable levels. Similar responses to aridity were previously documented in birds (Gilson et al. 2021) and insects (Lighton and Bartholomew 1988; Marron et al. 2003; Terblanche et al. 2010). The thread snake *Myriopholis macrorhyncha* did not reduce its metabolism despite being xeric and extremely small. We suspect its fossorial lifestyle in soils where water content is not as low as in other desert regions and its capacity to absorb moisture from its environment (Dubiner et al. 2024c) may render unnecessary physiological responses such as reduced RMR. In contrast, *T. yomtovi* and *C. sepsoides* have little access to water, giving mechanisms to reduce EWL an adaptive value. These mechanisms may have played a role in the widespread distribution of these species, their close relatives, and other small desert reptiles, across the Saharo‐Arabian desert.

As expected, all species increased their total EWL between dry air and humid air (approximating the natural conditions of xeric and mesic species, respectively). Xeric species had lower EWL than mesic species, as has been amply documented in previous studies (Bentley and Schmidt‐Nielsen 1966; Cox and Cox 2015; Dmi'el 2001; Gans et al. 1968; Mautz 1982). Furthermore, we found that the effect of dry air on xeric species’ EWL was weaker. These results hint at water‐conserving adaptations at work in desert species that commonly encounter dry air in their microhabitat. Interestingly, the EWL we observed for mesic species in humid (i.e., natural) conditions is equivalent to that of xeric species in dry (i.e., natural) conditions, suggesting that in nature, rates of evaporative water loss could be maintained the same across species from habitats with vastly different water availability. This could have implications for desert species' physiology and ecology, possibly requiring more efficient water acquisition from the diet and environment. EWL scaled allometrically with body size, with an exponent of 0.77, which is steeper than the exponent of 2/3 found in some previous comparative studies (Hlubeň et al. 2021; Le Galliard et al. 2021) and theoretically expected from a surface‐driven process such as cutaneous evaporation (Le Galliard et al. 2021). This may indicate that the respiratory component of EWL in our study animals might be more dominant than the cutaneous one since respiratory water loss is expected to scale as 3/4 (Le Galliard et al. 2021) rather than 2/3, as in our observations. We did not measure cutaneous and respiratory EWL separately and cannot refute or support this hypothesis. Alternatively, the surface area‐to‐volume ratio simply scaled more steeply than mathematically expected in our assemblage that contained thin and thick snakes and fully limbed and limb‐reduced lizard species. Differences in scale and skin structure (e.g., between a blind snake's barely‐keratinized scales and a skink's thick osteoderm) may also affect EWL rates and their allometry.

The physiological specialization demonstrated by our species supports the known evolutionary conservatism of affinity to habitat humidity in squamates (Cox and Cox 2015). Adaptations enabling water conservation are physiological but depend on selecting appropriate microhabitats with suitable humidity, and species that did not evolve to live in arid environments may face challenges if their habitats undergo changes in humidity or temperature due to climate change or land‐use change (Wu et al. 2025). Species from different habitats responded differently to dryness, with mesic species increasing more drastically in EWL. This result suggests that species in humid areas may be more vulnerable to increased water loss from climate warming (Mi et al. 2022) and aridification. This is especially true for very small species that are at a double risk of compromised water budgets (from increased EWL) and energy budgets (from increased RMR). The interdependence of EWL and RMR we describe here and the direct dependence of both on temperature further complicate this threat.

There are important methodological implications to our study. The influence of experimental humidity on RMR values may bias metabolic studies, especially those comparing the metabolic rates of species from different habitats. Various studies (e.g., Dubiner et al. 2023; Dupoué et al. 2017; Giacometti et al. 2022) have demonstrated lower metabolic rates in desert reptiles, but these data were based on measurements conducted in dry air (but see Lucchini et al. 2025). We found that xeric species’ RMRs were only lower than those of mesic species in dry air, but were similar in humid air. Thus, if only mesic species increase their metabolism (or xeric species reduce it) in response to dryness, then reported differences between habitats may be partly a differential response to the dry air rather than a difference in the metabolic rates experienced in nature. As for EWL, the diminished effect of dry air on xeric species may highlight a blind spot in biophysical models (e.g., NicheMapR; Kearney and Porter 2009). These models predict water loss from equations of heat and water exchange, and while species traits such as skin resistance can be set as parameters to determine rates of water exchange, little agency is usually given to adaptations regulating these exchanges under changing conditions. More importantly, the effects of humidity on RMR imply that some species’ energetic requirements are underestimated by these models under dry conditions. Biophysical models typically assume that humidity influences energy consumption not through any direct pathway, but indirectly, for example, through constraining evaporative cooling (Porter et al. 2024) or changing activity and food intake to balance out water loss (Kearney et al. 2013). A recent statement by Porter et al. (2024), *“if humidity affects endotherm thermoregulation, laboratory experiments that ignore humidity may not fully capture variation* […] *in the field,”* can certainly be applied to ectotherms as well, and to their metabolic and hydric regulation mechanisms. This holds true beyond vertebrate ectotherms, as studies have also found shifts in energy metabolism in response to dry conditions in various arthropod groups (e.g., scorpions: Kalra and Gefen 2012; cockroaches: Vrtar et al. 2018; beetles: Terblanche et al. 2010; ants: Lighton and Bartholomew 1988; flies: Gibbs 2002).

Environmental humidity has hitherto been an understudied facet of reptile ecophysiology, and our study demonstrates that the varied role it has on other physiological traits merits closer consideration. Primarily, the design and interpretation of future ecophysiological research needs to consider ambient humidity as a factor, for example, by controlling experimental humidity to match natural conditions. Alternatively, this could be addressed by considering possible effects of humidity during analysis and interpretation of dry‐air experiment results (or biophysical model predictions), but more research is needed before we can understand and predict the effects of humidity. First, it is important to uncover which of the differences between species are heritable and which are merely the effect of acclimation (Weaver et al. 2023). Future studies should also focus on the relationship between temperature and humidity to understand how these two key factors interplay to shape RMR (and EWL). Furthermore, the two habitats we compared do not span the full range of possible conditions: Tropical species inhabit more humid habitats than our “mesic” species and may therefore be even more susceptible to dryness. For example, the total EWL in the 0.45‐g tropical gecko *Sphaerodactylus macrolepis*, measured in 24°C dry air (Snyder 1975) was almost twice as high as the 0.51‐g *A. rueppellii* we tested under nearly identical conditions (even though the mass‐specific EWL of *A. rueppellii* was the highest of all our species). RMR responses to dry air might therefore be present at higher rates, or extend to larger body sizes, in tropical species. Since most of the world's reptiles inhabit areas with higher humidity than our study area, especially tropical regions (Meiri 2024), we predict most species worldwide to be even more susceptible to dryness and the patterns shown in this study to be yet more prevalent. Understanding these patterns would be crucial for predicting how species might respond to environmental changes and for managing habitats to protect their reptile biodiversity in the face of global changes.

## Supporting information




**File S1**:R code.

**Appendix 1–3**.: inz213016‐sup‐0003‐TableS1.pdf

**Supporting Table 1**: Raw data for all measurements conducted in this study. Individual measurements in the two replicates are in CO2 mL/min H2O mg/min, whereas RMR and EWL are their means (colored from to in each species) after converting to O2 uL/g/h and H2O mg/g/h.

**Supporting Table 2 A**: Raw microhabitat and site data measured opportunistically under shelters in the field where our study species were found. “Out” is a point outside the shelter (arbitrarily 20 cm south of it) B: Microhabitat and site data for the animals included in the experiment.

**Supporting Table 3** Species‐specific results of the GLMMs for RMR and EWL.

**Supporting Table 4** ValidaƟ on in two species of our CO2‐based results (showing higher RMR in dry air) against an O2‐based measure of RMR (using an Oxzilla O2 analyzer).

**Supporting Table 5**: Multi‐species results of the PGLMMs for RMR and EWL.
