# Supplementary material for: An Overlooked Habitat‐Dependent Link Between Metabolism and Water Loss in Reptiles
Source: Integr Zool. 2025 Jul 21;21(3):709–19. doi: 10.1111/1749-4877.13016 (PMC13164836; doi:10.1111/1749-4877.13016)
Supplement: Supplementary file 2 — Appendix 1–3.: inz213016‐sup‐0003‐TableS1.pdf [file INZ2-21-709-s002.docx]

**SUPPLEMENTARY MATERIALS**

**Appendix 1**

**a**) Images of the study species. Ty: *Tropiocolotes yomtovi*, Ss: *Stenodactylus sthenodactylus*, Ht: *Hemidactylus turcicus,* Ar: *Ablepharus rueppellii*, Cs: *Chalcides sepsoides*, Co: *Chalcides ocellatus,* Mm: *Myriopholis macrorhyncha*, Xs: *Xerotyphlops syriacus*, Ej: *Eryx jaculus,* Er: *Eirenis rothii,* Ld: *Lythorhynchus diadema*, Ed: *Eirenis decemlineatus.* Photos by Simon Jamison, Jonathan Ben Simon, and Eran Levin.

**b**) The phylogenetic tree used in our PGLMM analyses, subset from Zheng & Weins (2016), and each species’ mean body mass in our sample.

**
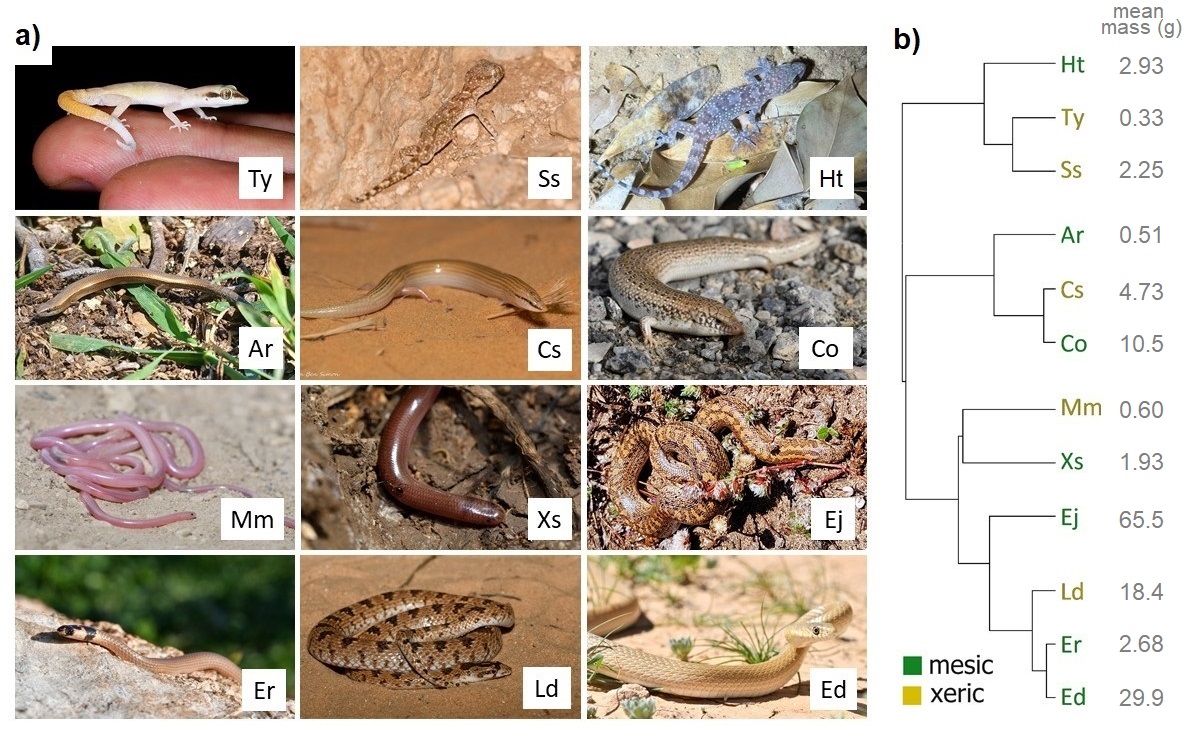
**

**Appendix 2**

Schematic diagram of the experimental setup.


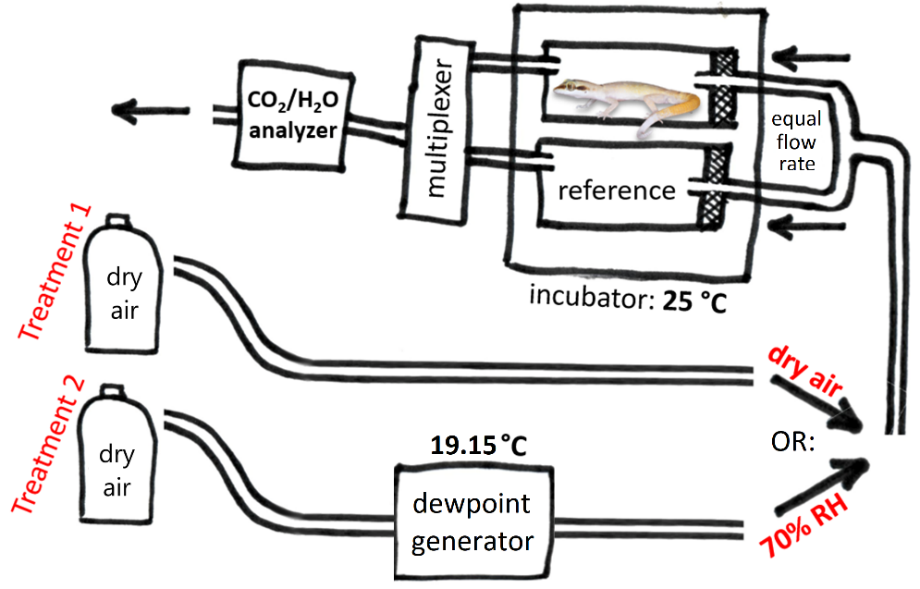


**Appendix 3**

Mean specific RMR (**a**) and EWL (**b**) for all species, under both treatments, based on **Table S1**. Significant differences between treatments (according to the GLMM) **: p < 0.01; ***: p < 0.001; *ns*: not significant.

**
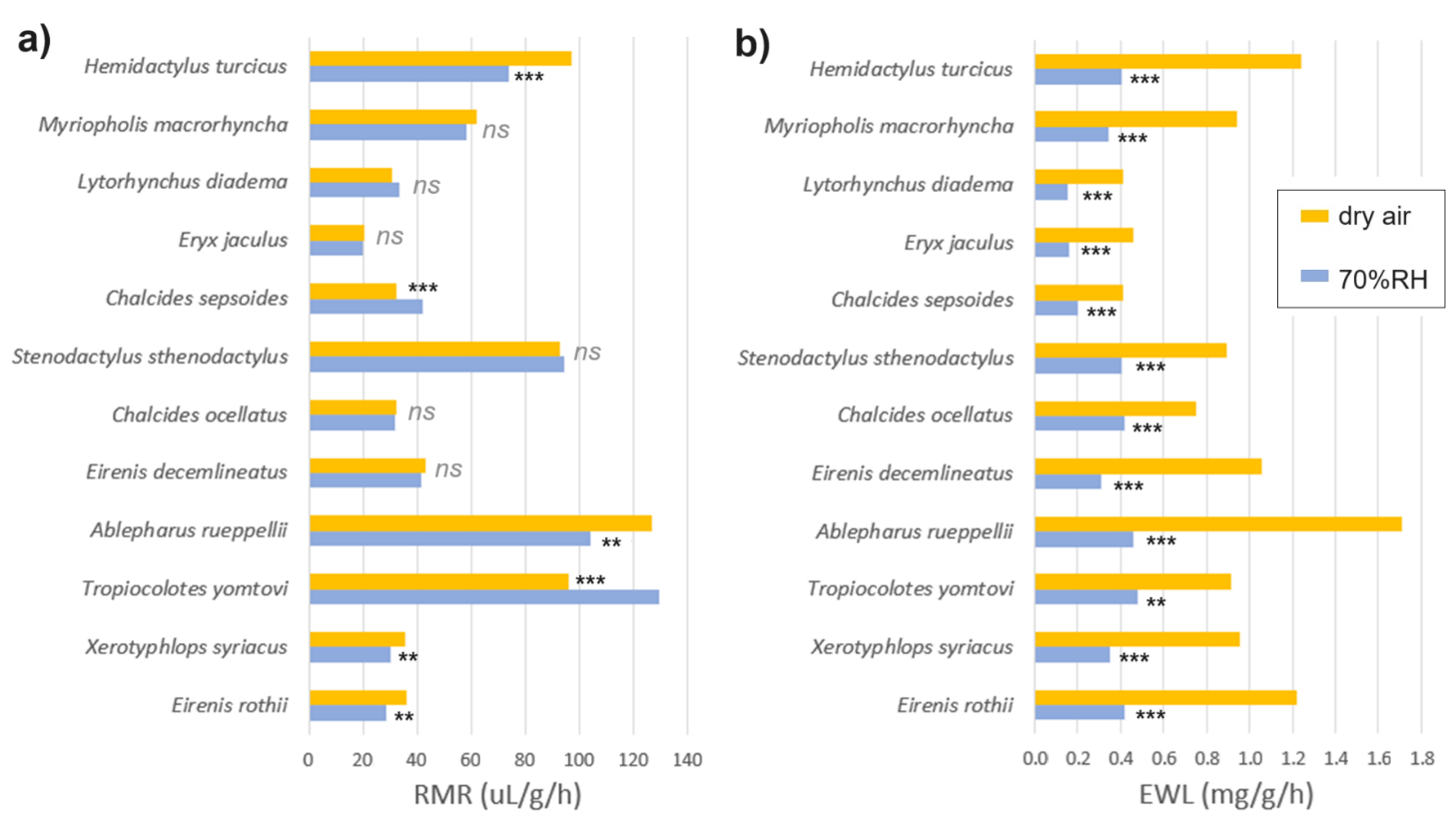
**
