# Supplementary material for: An Overlooked Habitat‐Dependent Link Between Metabolism and Water Loss in Reptiles
Source: Integr Zool. 2025 Jul 21;21(3):709–19. doi: 10.1111/1749-4877.13016 (PMC13164836; doi:10.1111/1749-4877.13016)
Supplement: Supplementary file 3 — Supporting Table 1: Raw data for all measurements conducted in this study. Individual measurements in the two replicates are in CO2 mL/min H2O mg/min, whereas RMR and EWL are their means (colored from to in each species) after converting to O2 uL/g/h and H2O mg/g/h. [file INZ2-21-709-s004.pdf]

Table S1: Raw data for all measurements conducted in this study. Individual measurements in the two replicates are in CO2 mL/min H2O mg/min, whereas RMR and EWL are their means (coloured from low to high in each species) after converting to O2 uL/g/hr and H2O mg/g/hr. "Blocks" were collected and measured (in random order) on the same occasion.

Species	ID	Mass	RMR: humid	RMR: dry	EWL: humid	EWL: dry	Replicate #1				Replicate #2						
							VCO2_humid	VCO2_dry	VH2O_humid	VH2O_dry	VCO2_humid	VCO2_dry	VH2O_humid	VH2O_dry	Block	Treatment_order	Collection site
Eirenis rothii	1	2.6	31.13516	30.96176	0.57735	1.30081	0.001286421	0.000959868	0.033904395	0.0588541	0.000872283	0.001186814	0.016132395	0.053883163	A	dry-humid, humid-dry	Jerusalem
Eirenis rothii	2	2.7	38.49072	43.86899	0.45678	1.26033	0.001506629	0.001098239	0.022122192	0.0508092	0.001264703	0.002060328	0.018987743	0.06262065	A	dry-humid, humid-dry	Jerusalem
Eirenis rothii	3	2.7	25.76558	34.83642	0.24578	1.00843	0.001106347	0.001163485	0.011127658	0.0491507	0.000748775	0.001344737	0.010992327	0.041607784	A	dry-humid, humid-dry	Jerusalem
Eirenis rothii	4	3.6	24.71372	32.20858	0.38232	1.16058	0.001096935	0.001532067	0.01639673	0.0641521	0.001275582	0.001559957	0.029481699	0.075117974	B	humid-dry, dry-humid	Jerusalem
Eirenis rothii	5	2.1	25.32141	31.15447	0.37723	1.08590	0.000583673	0.000577813	0.010161815	0.0257252	0.000834327	0.001166837	0.01624421	0.050287629	B	humid-dry, dry-humid	Jerusalem
Eirenis rothii	6	2.4	25.37775	45.04578	0.47218	1.52317	0.00078674	0.001752866	0.017743815	0.0623494	0.000837436	0.001130064	0.02003019	0.05950397	B	humid-dry, dry-humid	Jerusalem
	mean	2.683	**28.46739**	**36.34600**	**0.41860**	**1.22320**											
	SD	0.058	5.448248029	6.44392143	0.111735343	0.18236394											
							VCO2_humid	VCO2_dry	VH2O_humid	VH2O_dry	VCO2_humid	VCO2_dry	VH2O_humid	VH2O_dry	Block	Treatment_order	Collection site
Xerotyphlops syriacus	1	2.5	29.22913	42.27617	0.26234	0.96810	0.000705728	0.001377015	0.008555662	0.0395763	0.00124288	0.001441396	0.013305758	0.041099093	A	humid-dry, dry-humid	Jerusalem
Xerotyphlops syriacus	2	1.8	26.45030	31.99979	0.29621	1.03942	0.00052239	0.000693401	0.007628111	0.0353732	0.000747225	0.000842589	0.010144755	0.026992201	A	humid-dry, dry-humid	Jerusalem
Xerotyphlops syriacus	3	1.5	38.13222	35.34965	0.41087	1.03941	0.00065548	0.000655937	0.00898387	0.0257929	0.000869809	0.00075805	0.011559635	0.026177653	A	humid-dry, dry-humid	Jerusalem
Xerotyphlops syriacus	4	1.8	27.69788	39.05790	0.46405	0.98334	0.000795293	0.000840302	0.01139199	0.0262311	0.000534205	0.001034477	0.016450783	0.032769248	B	dry-humid, humid-dry	Jerusalem
Xerotyphlops syriacus	5	1.9	33.73948	36.22567	0.41425	0.84391	0.000733847	0.000911962	0.007881128	0.0273008	0.00097562	0.000923472	0.018354444	0.026146554	B	dry-humid, humid-dry	Jerusalem
Xerotyphlops syriacus	6	2.1	25.35728	27.17353	0.27250	0.84218	0.000604424	0.000780071	0.004737351	0.0408591	0.000815583	0.000741647	0.014337678	0.018093252	B	dry-humid, humid-dry	Jerusalem
	mean	1.933	**30.10105**	**35.34712**	**0.35337**	**0.95273**											
	SD	0.513	6.102652587	5.24089764	0.077841376	0.04117261											
							VCO2_humid	VCO2_dry	VH2O_humid	VH2O_dry	VCO2_humid	VCO2_dry	VH2O_humid	VH2O_dry	Block	Treatment_order	Collection site
Tropiocolotes yomtovi	1	0.37	107.92783	71.76062	0.28360	0.42030	0.000624953	0.000315357	0.001078587	0.003785	0.000439935	0.000392681	0.002419162	0.001398793	A	dry-humid, humid-dry	Ein Gedi
Tropiocolotes yomtovi	2	0.29	103.07935	55.52721	0.46973	1.26242	0.000420991	0.000144905	0.001144439	0.0058516	0.000376156	0.000284506	0.00339627	0.006351769	A	dry-humid, humid-dry	Ein Gedi
Tropiocolotes yomtovi	3	0.27	188.17503	130.37732	0.38296	0.57596	0.000709337	0.000501311	0.00157211	0.0024231	0.000645523	0.000437406	0.001874541	0.002760481	B	dry-humid, humid-dry	Lipa Gal
Tropiocolotes yomtovi	4	0.29	136.73070	119.39706	1.26009	0.84172	0.000493782	0.0004766	0.004884028	0.0042335	0.000563602	0.000446737	0.007296843	0.003903173	B	humid-dry, dry-humid	Lipa Gal
Tropiocolotes yomtovi	5	0.28	98.61454	105.02950	0.11422	1.73921	0.000325884	0.000375759	0.000884985	0.0079481	0.000410438	0.000408461	0.000181032	0.00828455	B	humid-dry, dry-humid	Lipa Gal
Tropiocolotes yomtovi	6	0.16	141.04664	93.98620	0.38396	0.64275	0.000332888	0.00019685	0.001471794	0.0016679	0.000268911	0.000204158	0.000576	0.001760104	B	humid-dry, dry-humid	Lipa Gal
	mean	0.277	**129.26235**	**96.01299**	**0.48243**	**0.91373**											
	SD	0.053	33.89793149	28.4267608	0.399905206	0.49783187											
							VCO2_humid	VCO2_dry	VH2O_humid	VH2O_dry	VCO2_humid	VCO2_dry	VH2O_humid	VH2O_dry	Block	Treatment_order	Collection site
Ablepharus rueppellii	1	0.26	77.46176	84.63511	0.56167	1.95110	0.0002261	0.00040629	0.003037904	0.0084548	0.000310968	0.000180513	0.001829876	0.00845478	A	dry-humid, humid-dry	Zichron
Ablepharus rueppellii	2	0.52	71.54419	75.29701	0.35668	1.82364	0.000546286	0.000667729	0.003249332	0.0209553	0.000445793	0.00037639	0.002933157	0.010654548	A	dry-humid, humid-dry	Zichron
Ablepharus rueppellii	3	0.61	110.05153	131.85129	config error	1.53925	0.000843712	0.001045919	NA (>limit)	0.0162805	0.00094646	0.001098862	NA (>limit)	0.015649052	B	humid-dry, dry-humid	Bajouriya
Ablepharus rueppellii	4	0.6	120.11894	167.28769	config error	2.44755	0.000842256	0.001360237	NA (>limit)	0.0103283	0.001079647	0.001316366	NA (>limit)	0.024475483	B	humid-dry, dry-humid	Bajouriya
Ablepharus rueppellii	5	0.47	152.19263	185.81477	config error	lost tail prior	0.00090485	0.001112762	NA (>limit)	NA (=1.67)	0.001002631	0.001216117	NA (>limit)	NA (=1.23)	B	humid-dry, dry-humid	Bajouriya
Ablepharus rueppellii	6	0.58	91.53220	115.23144	config error	1.68834	0.000687525	0.00088825	NA (>limit)	0.0120849	0.000728174	0.000893997	NA (>limit)	0.016320606	B	dry-humid, humid-dry	Bajouriya
	mean	0.507	**103.81688**	**126.68622**	**0.45917**	**1.71090**											
	SD	0.078	20.73614644	30.3176359	0.14494619	0.19327902											
							VCO2_humid	VCO2_dry	VH2O_humid	VH2O_dry	VCO2_humid	VCO2_dry	VH2O_humid	VH2O_dry	Block	Treatment_order	Collection site
Eirenis decemlineatus	1	13.5	44.05778	46.19717	config error	0.68295	0.009582703	0.009197466	NA (>limit)	0.2046677	0.006278099	0.007433514	NA (>limit)	0.102658905	A	humid-dry, dry-humid	Bajouriya
Eirenis decemlineatus	2	24.7	19.92715	24.84789	config error	0.36329	0.006843348	0.00864097	NA (>limit)	0.1559397	0.006282003	0.007725509	NA (>limit)	0.143172118	A	humid-dry, dry-humid	Bajouriya
Eirenis decemlineatus	3	40.5	41.95306	40.96633	0.20782	0.79229	0.03220775	0.02487393	0.244174381	0.8281067	0.01310155	0.01936971	0.036376321	0.241490668	B	humid-dry, dry-humid	Tel Aviv
Eirenis decemlineatus	4	47.1	25.78252	26.74014	0.11752	0.36808	0.02049732	0.02313263	0.102441356	0.3627216	0.01188552	0.01045299	0.082059292	0.215170148	B	dry-humid, humid-dry	Tel Aviv
Eirenis decemlineatus	5	23.7	75.90479	77.93161	0.13963	0.43936	0.02513952	0.02887376	0.05523138	0.1965642	0.02283231	0.02037902	0.055076269	0.150532717	C	dry-humid, humid-dry	Yokneam
	mean	29.90	**41.52506**	**43.33663**	**0.15499**	**0.52920**											
	SD	13.63	21.81089927	21.3769189	0.047067627	0.19647586											
							VCO2_humid	VCO2_dry	VH2O_humid	VH2O_dry	VCO2_humid	VCO2_dry	VH2O_humid	VH2O_dry	Block	Treatment_order	Collection site
Chalcides ocellatus	1	7.1	51.65965	45.26144	0.41975	0.52828	0.004859325	0.005643073	0.053133846	0.0368465	0.004921568	0.002926426	0.04620688	0.088179632	A	dry-humid, humid-dry	Yokneam
Chalcides ocellatus	2	8.1	31.56094	39.95777	0.12315	0.40797	0.003952994	0.00553518	0.016914752	0.0683403	0.002864168	0.003095698	0.016335265	0.041811647	A	dry-humid, humid-dry	Yokneam
Chalcides ocellatus	3	12.2	17.91001	20.90987	0.12185	0.21554	0.00310238	0.003375752	0.019423187	0.0220531	0.002724342	0.003426926	0.030127712	0.06559807	A	dry-humid, humid-dry	Yokneam
Chalcides ocellatus	4	12.1	29.74851	30.94811	0.15509	0.31499	0.004544826	0.005122084	0.022855175	0.059238	0.005054025	0.004863839	0.039699128	0.067807358	B	humid-dry, dry-humid	Yokneam
Chalcides ocellatus	5	12.6	25.73144	26.22272	defecated (twic	0.33378	0.003654034	0.004828203	NA (=7.37)	0.078731	0.004991731	0.003982631	NA (=4.34)	0.061456882	B	humid-dry, dry-humid	Yokneam
Chalcides ocellatus	6	10.6	33.24559	31.05786	0.22520	0.45588	0.005372823	0.004703506	0.048190162	0.083401	0.004024596	0.004075515	0.031379303	0.077676845	B	humid-dry, dry-humid	Yokneam
	mean	10.45	**31.64269**	**32.39296**	**0.20901**	**0.37607**											
	SD	2.7	16.97716233	12.8059241	0.171619876	0.15775196											
							VCO2_humid	VCO2_dry	VH2O_humid	VH2O_dry	VCO2_humid	VCO2_dry	VH2O_humid	VH2O_dry	Block	Treatment_order	Collection site
Stenodactylus sthenodactylus	1	2.0	86.59221	70.64329	0.26612	0.81009	0.002211083	0.002212998	0.006527133	0.0355937	0.002407168	0.001554644	0.011214105	0.018412043	A	humid-dry, dry-humid	Shivta
Stenodactylus sthenodactylus	2	4.0	153.90811	132.70688	0.36289	0.70345	0.009238103	0.00744498	0.013957888	0.058718	0.007178762	0.006710421	0.034426839	0.035074857	A	humid-dry, dry-humid	Shivta
Stenodactylus sthenodactylus	3	1.7	89.47637	110.68665	0.37191	0.72217	0.002917268	0.002788346	0.014124698	0.0186295	0.001138994	0.002229449	0.006950473	0.022293384	A	humid-dry, dry-humid	Shivta
Stenodactylus sthenodactylus	4	2.0	67.73499	75.15735	0.59259	1.08520	0.002443952	0.002266447	0.028494227	0.041606	0.001168581	0.001741945	0.011011507	0.030740569	B	dry-humid, humid-dry	Shivta

Stenodactylus sthenodactylus	5	2.0	61.19901	97.84614	0.41025	1.25799	0.001744652	0.003283713	0.01688003	0.0566782	0.001519295	0.001934748	0.010469713	0.027187569	B	dry-humid, humid-dry	Shivta
Stenodactylus sthenodactylus	6	1.8	108.42758	69.77558	0.44350	0.77817	0.00387602	0.001726692	0.018373292	0.0230887	0.001328504	0.001622536	0.008236505	0.023601398	B	dry-humid, humid-dry	Shivta
	mean	2.25	**94.55638**	**92.80265**	**0.40787**	**0.89284**											
	SD	0.867	38.05959919	31.4649324	0.05864895	0.05693864											
							VCO2_humid	VCO2_dry	VH2O_humid	VH2O_dry	VCO2_humid	VCO2_dry	VH2O_humid	VH2O_dry	Block	Treatment_order	Collection site
Chalcides sepsoides	1	5.7	41.35791	31.48797	0.08961	0.16165	0.002728386	0.002333683	0.008144098	0.0137429	0.003558016	0.002452489	0.008881696	0.016971392	A	humid-dry, dry-humid	Ashdod
Chalcides sepsoides	2	6.1	49.28222	39.09920	0.10743	0.21516	0.003751626	0.003496749	0.009977969	0.0238315	0.004264948	0.002863387	0.011866735	0.019916843	A	humid-dry, dry-humid	Ashdod
Chalcides sepsoides	3	5.1	44.33071	27.60457	0.12157	0.19563	0.00287986	0.001990452	0.010659054	0.01647	0.003149116	0.00176377	0.010008255	0.016787879	A	humid-dry, dry-humid	Ashdod
Chalcides sepsoides	4	3.9	43.29347	38.61773	0.11053	0.20661	0.001844089	0.001799125	0.007578644	0.0108731	0.002658432	0.002217119	0.006790653	0.015986597	B	dry-humid, humid-dry	Beer Milka
Chalcides sepsoides	5	2.6	46.39327	36.41755	0.09750	0.17064	0.001161009	0.001286498	0.003982567	0.0073243	0.002055591	0.001238452	0.004467208	0.007464405	B	dry-humid, humid-dry	Beer Milka
Chalcides sepsoides	6	5	28.58621	22.27304	0.07913	0.29351	0.00174487	0.001370439	0.001553316	0.0273349	0.002066624	0.0015993	0.011634923	0.021582816	C	dry-humid, humid-dry	Ashdod
	mean	4.73	**42.20730**	**32.58334**	**0.10096**	**0.20720**											
	SD	0.50	4.003116921	5.84719128	0.016016828	0.02707441											
							VCO2_humid	VCO2_dry	VH2O_humid	VH2O_dry	VCO2_humid	VCO2_dry	VH2O_humid	VH2O_dry	Block	Treatment_order	Collection site
Eryx jaculus	1	58.1	24.92631	27.63673	0.06311	0.21493	0.02190149	0.02469483	0.076643014	0.291491	0.01671768	0.01812368	0.045581433	0.208122669	A	humid-dry-dry-humid	Tel Aviv
Eryx jaculus	2	80.3	12.34296	17.21674	0.07085	defecated (tw	0.01361302	0.02318412	0.126836303	NA (=16.67)	0.01281738	0.01368266	0.062806331	NA (=12.97)	A	humid-dry-dry-humid	Tel Aviv
Eryx jaculus	3	99.1	34.93939	28.01755	0.17327	0.23255	0.05272391	0.03543705	0.47663975	0.4533224	0.03960924	0.038604	0.095728642	0.384089859	A	humid-dry-dry-humid	Tel Aviv
Eryx jaculus	4	46.5	15.37010	14.86555	0.04635	0.19608	0.008154689	0.0108692	0.032741006	0.1564436	0.01090424	0.007564076	0.039105449	0.151963751	B	dry-humid-humid-dry	Yokneam
Eryx jaculus	5	43.7	11.40504	12.49391	0.04350	0.19659	0.006928849	0.008603663	0.023929626	0.1673379	0.00636182	0.005955911	0.039440433	0.143181941	B	dry-humid-humid-dry	Yokneam
	mean	65.54	**19.79676**	**20.43492**	**0.07942**	**0.23091**											
	SD	23.66	10.01482438	6.83190627	0.053690543	0.02931312											
							VCO2_humid	VCO2_dry	VH2O_humid	VH2O_dry	VCO2_humid	VCO2_dry	VH2O_humid	VH2O_dry	Block	Treatment_order	Collection site
Lytorhynchus diadema	1	20.1	28.39708	33.88265	0.08922	0.28944	0.00551697	0.00662378	0.035999118	0.1277443	0.009703867	0.01153732	0.023777232	0.066182884	A	humid-dry-dry-humid	Ashdod
Lytorhynchus diadema	2	27.6	29.05271	33.05605	0.06556	0.32021	0.007553505	0.00834192	0.038208413	0.2051167	0.01382929	0.01598733	0.022108762	0.089477167	A	humid-dry-dry-humid	Ashdod
Lytorhynchus diadema	3	28.4	36.53589	40.28031	0.05917	0.27014	0.010094375	0.009694335	0.041267391	0.1734846	0.01757547	0.02081129	0.014751044	0.082249069	A	humid-dry-dry-humid	Ashdod
Lytorhynchus diadema	4	6	51.34046	28.07560	0.11715	0.09234	0.005524356	0.002364083	0.013275548	0.0090535	0.002690118	0.002128013	0.010154778	0.009413724	B	dry-humid-humid-dry	Beer Milka
Lytorhynchus diadema	5	10	20.85153	17.43533	0.04986	0.05729	0.003034766	0.002338574	0.0054084	0.0089646	0.002525643	0.002310848	0.0112124	0.010131748	B	dry-humid-humid-dry	Beer Milka
	mean	18.42	**33.23554**	**30.54599**	**0.07619**	**0.20588**											
	SD	4.579	11.54286401	8.51737814	0.027125724	0.12160942											
							VCO2_humid	VCO2_dry	VH2O_humid	VH2O_dry	VCO2_humid	VCO2_dry	VH2O_humid	VH2O_dry	Block	Treatment_order	Collection site
Myriopholis macrorhyncha	1	0.84	47.65185	47.05888	0.47065	1.00823	0.000220725	0.000527059	0.005295209	0.0141152	0.000846677	NA (agitated)	0.007883122	NA (agitated)	A	dry-humid-humid-dry	Ein Gedi
Myriopholis macrorhyncha	2	0.52	55.41987	71.61389	0.32412	1.17776	0.000444561	0.000505665	0.003794043	0.0091563	0.000323928	0.000487381	0.001824077	0.011258117	B	humid-dry-dry-humid	Gilboa
Myriopholis macrorhyncha	3	0.77	47.31188	53.88128	0.22087	0.60378	0.000605303	0.000585776	0.003453731	0.0061599	0.000366167	0.000520587	0.002215327	0.00933697	B	humid-dry-dry-humid	Gilboa
Myriopholis macrorhyncha	4	0.48	55.47433	45.67727	0.41347	0.64684	0.000383485	0.000325474	0.003348138	0.0037254	0.000326586	0.000259195	0.003267319	0.006623989	B	dry-humid-humid-dry	Gilboa
Myriopholis macrorhyncha	5	0.39	84.32344	91.24117	0.30180	1.27177	0.000459833	0.000584531	0.001370301	0.0097245	0.000417131	0.000364377	0.00255305	0.006808511	B	dry-humid-humid-dry	Gilboa
	mean	0.60	**58.03627**	**61.89450**	**0.34618**	**0.94167**											
	SD	0.168	15.22558111	19.3842692	0.097698281	0.3042321											
							VCO2_humid	VCO2_dry	VH2O_humid	VH2O_dry	VCO2_humid	VCO2_dry	VH2O_humid	VH2O_dry	Block	Treatment_order	Collection site
Hemidactylus turcicus	1	3.3	75.14028	119.55601	0.52153	0.86231	0.003259442	0.005272691	0.02868418	0.0449206	0.003352903	0.005248238	NA (=3.3)	0.049933683	A	dry-humid-humid-dry	Tel Aviv
Hemidactylus turcicus	2	3.6	88.13219	81.19008	0.20797	0.80977	0.00422693	0.004098906	0.014219739	0.0328849	0.00423376	0.003695342	0.010737109	0.064286882	A	dry-humid-humid-dry	Tel Aviv
Hemidactylus turcicus	3	3	81.67350	98.24179	0.57452	0.89712	0.00331316	0.003084522	0.029685675	0.0624275	0.00322072	0.004774821	0.027766805	0.027284039	A	dry-humid-humid-dry	Tel Aviv
Hemidactylus turcicus	4	3.3	59.58145	83.08481	0.24157	1.47127	0.002867334	0.004656866	0.01916725	0.0926549	0.002375834	0.002654597	0.007405973	0.069184646	B	humid-dry-dry-humid	Tel Aviv
Hemidactylus turcicus	5	1.5	55.26530	91.02460	0.69265	1.57194	0.001040427	0.001841102	0.018673205	0.0380519	0.001170185	0.001799882	0.015959391	0.040544948	B	humid-dry-dry-humid	Tel Aviv
Hemidactylus turcicus	6	2.9	84.94999	108.21107	0.30260	1.84259	0.00329589	0.003864929	0.005289002	NA (=4.55)	0.003273576	0.004503394	0.023962667	0.089058319	B	humid-dry-dry-humid	Tel Aviv
	mean	2.933	**74.12379**	**96.88473**	**0.40566**	**1.24250**											
	SD	0.3	13.70106199	14.9346282	0.222463658	0.44092198											
