# Supplementary material for: An Overlooked Habitat‐Dependent Link Between Metabolism and Water Loss in Reptiles
Source: Integr Zool. 2025 Jul 21;21(3):709–19. doi: 10.1111/1749-4877.13016 (PMC13164836; doi:10.1111/1749-4877.13016)
Supplement: Supplementary file 4 — Supporting Table 2 A: Raw microhabitat and site data measured opportunistically under shelters in the field where our study species were found. “Out” is a point outside the shelter (arbitrarily 20 cm south of it) B: Microhabitat and site data for the animals included in the experiment. [file INZ2-21-709-s007.pdf]

Table S2 A: Raw microhabitat and site data measured opportunistically under shelters in the field where our study species were found. “Out” is a point outside the shelter (arbitrarily 20cm south of it) **B**: Microhabitat and site data for the animals included in the experiment.

Table S2a:

species	ID	date	locality	coordinates	RH (%)	Tsub (°C)	VPD	out RH	out Tsub	rock dimensions (cm)	soil water content (cm^3/cm^3, -10kPa 0-5cm depth)
Ablepharus rueppellii	A1	01/04/2023	Yokneam	lat 32.641, long 35.094	70.3	25.7	1.0	38.0	24.8	20x11	NA
Ablepharus rueppellii	A2	02/04/2023	Jerusalem	lat 31.738, long 35.207	64.5	28.9	1.4	62.2	30.3	41x33	NA
Ablepharus rueppellii	A3	02/04/2023	Jerusalem	lat 31.738, long 35.207	84.6	28	0.6	59.4	25.6	36x15	NA
Ablepharus rueppellii	A4	23/04/2023	Yokneam	lat 32.641, long 35.094	69.8	25.7	1.0	55.9	41.4	47x37	NA
Ablepharus rueppellii	A5	23/04/2023	Yokneam	lat 32.641, long 35.094	87.8	27.2	0.4	59.2	37.9	32x29	NA
Ablepharus rueppellii	A6	12/06/2023	Golan	near Qatsrin	76.4	29.8	1.0	55.4	36.3	73x41	NA
Ablepharus rueppellii	A7	12/06/2023	Golan	near Qatsrin	67.4	27.4	1.2	46.5	27.5	51x26	NA
Ablepharus rueppellii	A8	06/04/2024	Yokneam	lat 32.641, long 35.094	60.3	29.3	1.6	42.1	33.0	30x17	NA
Ablepharus rueppellii	A9	11/04/2024	Yokneam	lat 32.641, long 35.094	71.9	28.7	1.1	53.4	34.5	27x14	NA
Chalcides sepsoides	C1	07/11/2023	Ashdod	lat 31.774, long 34.653	64.8	35.5	2.0	38.4	39.6	NA	NA
Chalcides sepsoides	C2	07/11/2023	Ashdod	lat 31.774, long 34.653	50.2	35.7	2.9	46.7	35.3	NA	NA
Chalcides ocellatus	O1	28/03/2024	Ein Gedi	lat 31.467, long 35.392	69.3	24.1	0.9	54.6	24.9	46x35	NA
Chalcides ocellatus	O2	28/03/2024	Ein Gedi	lat 31.467, long 35.392	78.1	24.5	0.7	40.2	30.1	32x21	NA
Chalcides ocellatus	O3	06/04/2024	Yokneam	lat 32.641, long 35.094	78.4	21.2	0.5	57.1	26.4	37x22	NA
Chalcides ocellatus	O4	06/04/2024	Yokneam	lat 32.641, long 35.094	77.3	27.6	0.8	62.7	29.7	30x53	NA
Chalcides ocellatus	O5	06/04/2024	Yokneam	lat 32.641, long 35.094	47.5	27.7	1.9	41.5	30.4	46x33	NA
Chalcides ocellatus	O6	06/04/2024	Yokneam	lat 32.641, long 35.094	66.5	28.8	1.3	42.7	39.7	45x15	NA
Chalcides ocellatus	O7	06/04/2024	Yokneam	lat 32.641, long 35.094	67.7	31.1	1.5	53.6	43.3	31x27	NA
Chalcides ocellatus	O8	11/04/2024	Yokneam	lat 32.641, long 35.094	88.8	30.2	0.5	77.8	29.5	27x24	NA
Chalcides ocellatus	O9	17/04/2024	Ein Gedi	lat 31.467, long 35.392	60.8	28.1	1.5	49.8	29.3	34x28	NA
Chalcides ocellatus	O10	29/04/2024	En Al Assad	lat 32.932, long 35.420	72.9	31.7	1.3	57.5	31.9	20x13	NA
Eirenis rothii	E1	01/04/2023	Yokneam	lat 32.641, long 35.094	67.3	25.3	1.1	40.1	23.7	25x18	NA
Eirenis rothii	E2	01/04/2023	Yokneam	lat 32.641, long 35.094	72.9	26.0	0.9	43.3	25.4	41x27	NA
Eirenis rothii	E3	02/04/2023	Jerusalem	lat 31.738, long 35.207	75.4	26.0	0.8	43.6	29.3	36x17	NA
Eirenis rothii	E4	02/04/2023	Jerusalem	lat 31.738, long 35.207	83.3	25.2	0.5	74.4	27.5	28x21	NA
Eirenis rothii	E5	02/04/2023	Jerusalem	lat 31.738, long 35.207	81.3	31.7	0.9	50.7	27.7	29x25	NA
Eirenis rothii	E6	02/04/2023	Jerusalem	lat 31.738, long 35.207	84.6	28.0	0.6	59.4	25.6	36x15	NA
Eirenis rothii	E7	23/04/2023	Yokneam	lat 32.641, long 35.094	77.1	32.1	1.1	38.8	35.1	24x19	NA

Eirenis rothii	E8	01/05/2023	Jerusalem	lat 31.738, long 35.207	74.3	24.1	0.8	51.6	36.9	50x25	NA
Eirenis rothii	E9	01/05/2023	Jerusalem	lat 31.738, long 35.207	76.4	25.7	0.8	41.9	43.8	33x19	NA
Eirenis rothii	E10	01/05/2023	Jerusalem	lat 31.738, long 35.207	74.6	36.1	1.5	51.0	41.6	22x21	NA
Eirenis rothii	E11	01/05/2023	Jerusalem	lat 31.738, long 35.207	73.8	33.2	1.3	37.5	52.2	47x33	NA
Eirenis decemlineatus	D1	11/04/2024	Yokneam	lat 32.641, long 35.094	78.3	26.5	0.8	67.2	34.3	41x28	NA
Eryx jaculus	J1	11/04/2024	Yokneam	lat 32.641, long 35.095	70.1	24.7	0.9	64.8	35.1	92x34	NA
Eryx jaculus	J2	11/04/2024	Yokneam	lat 32.641, long 35.096	74.9	27.5	0.9	80.2	30.2	76x22	NA
Hemidactylus turcicus	H1	04/08/2024	Tel Aviv	lat 32.114, long 34.809	57.3	31.6	2.0	60.7	31.6	NA	NA
Hemidactylus turcicus	H2	04/08/2024	Tel Aviv	lat 32.114, long 34.809	55.2	33.8	2.4	53.2	35.9	NA	NA
Hemidactylus turcicus	H3	04/08/2024	Tel Aviv	lat 32.114, long 34.809	52.9	34.7	2.6	55.1	35.0	NA	NA
Hemidactylus turcicus	H4	05/08/2024	Tel Aviv	lat 32.114, long 34.809	66.8	24.4	1.0	70.1	24.8	NA	NA
Hemidactylus turcicus	H5	05/08/2024	Tel Aviv	lat 32.114, long 34.809	66.8	24.4	1.0	70.1	24.8	NA	NA
Hemidactylus turcicus	H6	21/08/2024	Tel Aviv	lat 32.114, long 34.809	70.1	30.3	1.3	67.4	29.8	NA	NA
Hemidactylus turcicus	H7	21/08/2024	Tel Aviv	lat 32.114, long 34.809	68.2	31.2	1.4	64.7	32.5	NA	NA
Hemidactylus turcicus	H8	21/08/2024	Tel Aviv	lat 32.114, long 34.809	68.2	31.2	1.4	64.7	32.5	NA	NA
Hemidactylus turcicus	H9	21/08/2024	Tel Aviv	lat 32.114, long 34.809	74.4	30.6	1.1	70.7	32.1	13x24	NA
Tropiocolotes yomtovi	T2	03/04/2023	Ein Gedi	lat 31.467, long 35.392	29.7	37.0	4.4	21.1	39.8	17x14	NA
Tropiocolotes yomtovi	T3	06/05/2023	Lipa Gal	lat 30.822, long 34.744	26.6	33.5	3.8	20.9	36.2	16x14	NA
Tropiocolotes yomtovi	T4	06/05/2023	Lipa Gal	lat 30.822, long 34.744	35.5	30.1	2.8	24.5	34.5	26x24	NA
Tropiocolotes yomtovi	T5	06/05/2023	Lipa Gal	lat 30.822, long 34.744	35.4	35.9	3.8	26.2	40.8	33x22	NA
Tropiocolotes yomtovi	T6	06/05/2023	Lipa Gal	lat 30.822, long 34.744	20.8	37.4	5.1	17.8	42.5	25x12	NA
Xerotyphlops syriacus	X1	02/04/2023	Jerusalem	lat 31.738, long 35.207	69.8	28.3	1.2	49.9	27.4	38x30	NA
Xerotyphlops syriacus	X2	02/04/2023	Jerusalem	lat 31.738, long 35.207	80.9	27.5	0.7	56.6	27.4	48x31	NA
Xerotyphlops syriacus	X3	02/04/2023	Jerusalem	lat 31.738, long 35.207	79.4	28.7	0.8	62.2	29.8	25x19	NA
Xerotyphlops syriacus	X4	02/04/2023	Jerusalem	lat 31.738, long 35.207	78.1	28.7	0.9	48.1	31.4	24x16	NA
Xerotyphlops syriacus	X5	16/04/2023	Netanya	lat 32.331, long 34.876	99.0	NA		99.0	NA	NA	NA
Xerotyphlops syriacus	X6	16/04/2023	Netanya	lat 32.331, long 34.876	99.0	NA		99.0	NA	NA	NA
Xerotyphlops syriacus	X7	16/04/2023	Netanya	lat 32.331, long 34.876	99.0	NA		99.0	NA	NA	NA
Xerotyphlops syriacus	X8	12/06/2023	Golan	near Qatsrin	68.7	27.2	1.1	51.4	32.8	50x27	NA

Table S2b:

species	ID	date	locality	coordinates	RH (%)	Tsub (°C)	VPD	out RH	out Tsub	rock dimensions (cm)	soil water content (cm^3/cm^3, -10kPa at 0-5cm depth)
Ablepharus rueppellii	Ar1	yearly mean	Zichron	lat 32.553, long 34.972	NA	NA	NA	NA	NA	NA	0.368
Ablepharus rueppellii	Ar2	yearly mean	Zichron	lat 32.553, long 34.972	NA	NA	NA	NA	NA	NA	0.368
Ablepharus rueppellii	Ar3	yearly mean	Bajouriya	lat 32.863, long 35.786	NA	NA	NA	NA	NA	NA	0.357
Ablepharus rueppellii	Ar4	yearly mean	Bajouriya	lat 32.863, long 35.786	NA	NA	NA	NA	NA	NA	0.357
Ablepharus rueppellii	Ar5	yearly mean	Bajouriya	lat 32.863, long 35.786	NA	NA	NA	NA	NA	NA	0.357
Ablepharus rueppellii	Ar6	yearly mean	Bajouriya	lat 32.863, long 35.786	NA	NA	NA	NA	NA	NA	0.357
Chalcides ocellatus	Co1	yearly mean	Yokneam	lat 32.641, long 35.095	NA	NA	NA	NA	NA	NA	0.356
Chalcides ocellatus	Co2	yearly mean	Yokneam	lat 32.641, long 35.095	NA	NA	NA	NA	NA	NA	0.356
Chalcides ocellatus	Co3	yearly mean	Yokneam	lat 32.641, long 35.095	NA	NA	NA	NA	NA	NA	0.356
Chalcides ocellatus	Co4	yearly mean	Yokneam	lat 32.641, long 35.095	NA	NA	NA	NA	NA	NA	0.356
Chalcides ocellatus	Co5	yearly mean	Yokneam	lat 32.641, long 35.095	NA	NA	NA	NA	NA	NA	0.356
Chalcides ocellatus	Co6	yearly mean	Yokneam	lat 32.641, long 35.095	NA	NA	NA	NA	NA	NA	0.356
Chalcides sepsoides	Cs1	yearly mean	Ashdod	lat 31.774, long 34.653	NA	NA	NA	NA	NA	NA	0.324
Chalcides sepsoides	Cs2	yearly mean	Ashdod	lat 31.774, long 34.653	NA	NA	NA	NA	NA	NA	0.324
Chalcides sepsoides	Cs3	yearly mean	Ashdod	lat 31.774, long 34.653	NA	NA	NA	NA	NA	NA	0.324
Chalcides sepsoides	Cs4	yearly mean	Beer Milka	lat 30.926, long 34.403	NA	NA	NA	NA	NA	NA	0.316
Chalcides sepsoides	Cs5	yearly mean	Beer Milka	lat 30.926, long 34.403	NA	NA	NA	NA	NA	NA	0.316
Chalcides sepsoides	Cs6	yearly mean	Ashdod	lat 31.7742, long 34.653	NA	NA	NA	NA	NA	NA	0.324
Eirenis rothii	Er1	yearly mean	Jerusalem	lat 31.738, long 35.207	NA	NA	NA	NA	NA	NA	0.36
Eirenis rothii	Er2	yearly mean	Jerusalem	lat 31.738, long 35.207	NA	NA	NA	NA	NA	NA	0.36
Eirenis rothii	Er3	yearly mean	Jerusalem	lat 31.738, long 35.207	NA	NA	NA	NA	NA	NA	0.36
Eirenis rothii	Er4	yearly mean	Jerusalem	lat 31.738, long 35.207	NA	NA	NA	NA	NA	NA	0.36
Eirenis rothii	Er5	yearly mean	Jerusalem	lat 31.738, long 35.207	NA	NA	NA	NA	NA	NA	0.36
Eirenis rothii	Er6	yearly mean	Jerusalem	lat 31.738, long 35.207	NA	NA	NA	NA	NA	NA	0.36
Eirenis decemlineatus	Ed1	yearly mean	Bajouriya	lat 32.863, long 35.786	NA	NA	NA	NA	NA	NA	0.357
Eirenis decemlineatus	Ed2	yearly mean	Bajouriya	lat 32.863, long 35.786	NA	NA	NA	NA	NA	NA	0.357
Eirenis decemlineatus	Ed3	yearly mean	Tel Aviv	lat 32.114, long 34.809	NA	NA	NA	NA	NA	NA	NA
Eirenis decemlineatus	Ed4	yearly mean	Tel Aviv	lat 32.114, long 34.809	NA	NA	NA	NA	NA	NA	NA
Eirenis decemlineatus	Ed5	yearly mean	Yokneam	lat 32.641, long 35.095	NA	NA	NA	NA	NA	NA	0.356
Eryx jaculus	Ej1	yearly mean	Tel Aviv	lat 32.114, long 34.809	NA	NA	NA	NA	NA	NA	NA

Eryx jaculus	Ej2	yearly mean	Tel Aviv	lat 32.114, long 34.809	NA	NA	NA	NA	NA	NA	NA
Eryx jaculus	Ej3	yearly mean	Tel Aviv	lat 32.114, long 34.809	NA	NA	NA	NA	NA	NA	NA
Eryx jaculus	Ej4	yearly mean	Yokneam	lat 32.641, long 35.095	NA	NA	NA	NA	NA	NA	0.356
Eryx jaculus	Ej5	yearly mean	Yokneam	lat 32.641, long 35.095	NA	NA	NA	NA	NA	NA	0.356
Hemidactylus turcicus	Ht1	yearly mean	Tel Aviv	lat 32.114, long 34.809	NA	NA	NA	NA	NA	NA	NA
Hemidactylus turcicus	Ht2	yearly mean	Tel Aviv	lat 32.114, long 34.809	NA	NA	NA	NA	NA	NA	NA
Hemidactylus turcicus	Ht3	yearly mean	Tel Aviv	lat 32.114, long 34.809	NA	NA	NA	NA	NA	NA	NA
Hemidactylus turcicus	Ht4	yearly mean	Tel Aviv	lat 32.114, long 34.809	NA	NA	NA	NA	NA	NA	NA
Hemidactylus turcicus	Ht5	yearly mean	Tel Aviv	lat 32.114, long 34.809	NA	NA	NA	NA	NA	NA	NA
Hemidactylus turcicus	Ht6	yearly mean	Tel Aviv	lat 32.114, long 34.809	NA	NA	NA	NA	NA	NA	NA
Lytorhynchus diadema	Ld1	yearly mean	Ashdod	lat 31.774, long 34.653	NA	NA	NA	NA	NA	NA	0.324
Lytorhynchus diadema	Ld2	yearly mean	Ashdod	lat 31.774, long 34.653	NA	NA	NA	NA	NA	NA	0.324
Lytorhynchus diadema	Ld3	yearly mean	Ashdod	lat 31.774, long 34.653	NA	NA	NA	NA	NA	NA	0.324
Lytorhynchus diadema	Ld4	yearly mean	Beer Milka	lat 30.926, long 34.403	NA	NA	NA	NA	NA	NA	0.316
Lytorhynchus diadema	Ld5	yearly mean	Beer Milka	lat 30.926, long 34.403	NA	NA	NA	NA	NA	NA	0.316
Myriopholis macrorhyncha	Mm1	yearly mean	Ein Gedi	lat 31.467, long 35.3923	NA	NA	NA	NA	NA	NA	0.325
Myriopholis macrorhyncha	Mm2	yearly mean	Gilboa	lat 32.509, long 35.422	NA	NA	NA	NA	NA	NA	0.364
Myriopholis macrorhyncha	Mm3	yearly mean	Gilboa	lat 32.509, long 35.422	NA	NA	NA	NA	NA	NA	0.364
Myriopholis macrorhyncha	Mm4	yearly mean	Gilboa	lat 32.509, long 35.422	NA	NA	NA	NA	NA	NA	0.364
Myriopholis macrorhyncha	Mm5	yearly mean	Gilboa	lat 32.509, long 35.422	NA	NA	NA	NA	NA	NA	0.364
Tropiocolotes yomtovi	Ty1	yearly mean	Ein Gedi	lat 31.467, long 35.392	NA	NA	NA	NA	NA	NA	0.325
Tropiocolotes yomtovi	Ty2	yearly mean	Ein Gedi	lat 31.467, long 35.392	NA	NA	NA	NA	NA	NA	0.325
Tropiocolotes yomtovi	Ty3	yearly mean	Lipa Gal	lat 30.822, long 34.744	NA	NA	NA	NA	NA	NA	0.312
Tropiocolotes yomtovi	Ty4	yearly mean	Lipa Gal	lat 30.822, long 34.744	NA	NA	NA	NA	NA	NA	0.312
Tropiocolotes yomtovi	Ty5	yearly mean	Lipa Gal	lat 30.822, long 34.744	NA	NA	NA	NA	NA	NA	0.312
Tropiocolotes yomtovi	Ty6	yearly mean	Lipa Gal	lat 30.822, long 34.744	NA	NA	NA	NA	NA	NA	0.312
Xerotyphlops syriacus	Xs1	yearly mean	Jerusalem	lat 31.738, long 35.207	NA	NA	NA	NA	NA	NA	0.36
Xerotyphlops syriacus	Xs2	yearly mean	Jerusalem	lat 31.738, long 35.207	NA	NA	NA	NA	NA	NA	0.36
Xerotyphlops syriacus	Xs3	yearly mean	Jerusalem	lat 31.738, long 35.207	NA	NA	NA	NA	NA	NA	0.36
Xerotyphlops syriacus	Xs4	yearly mean	Jerusalem	lat 31.738, long 35.207	NA	NA	NA	NA	NA	NA	0.36
Xerotyphlops syriacus	Xs5	yearly mean	Jerusalem	lat 31.738, long 35.207	NA	NA	NA	NA	NA	NA	0.36
Xerotyphlops syriacus	Xs6	yearly mean	Jerusalem	lat 31.738, long 35.207	NA	NA	NA	NA	NA	NA	0.36
