# Supplementary material for: An Overlooked Habitat‐Dependent Link Between Metabolism and Water Loss in Reptiles
Source: Integr Zool. 2025 Jul 21;21(3):709–19. doi: 10.1111/1749-4877.13016 (PMC13164836; doi:10.1111/1749-4877.13016)
Supplement: Supplementary file 5 — Supporting Table 3 Species‐specific results of the GLMMs for RMR and EWL. [file INZ2-21-709-s003.pdf]

Table S3: Species-specific results of the GLMMs for RMR and EWL. The "effect" is of dry air relative to humid air. "CI" is the 95% confidence interval.

		RMR					EWL				
**Species**	***n***	**Effect**	**upper CI**	**lower CI**	***t***	***p***	**Effect**	**upper CI**	**lower CI**	***t***	***p***
Stenodactylus sthenodactylus	6	1.021232	1.830245	0.569823	−4.26	0.829	2.241914	4.57529	1.098549	2.71	**<0.001**
Tropiocolotes yomtovi	6	0.730994	1.135951	0.470401	0.22	**<0.001**	2.016574	9.556294	0.425538	6.79	**0.007**
Hemidactylus turcicus	6	1.331745	1.96826	0.901072	4.4	<0.001	2.999963	9.152252	0.98334	5.91	**<0.001**
Ablepharus rueppellii	6	1.191544	1.773988	0.800331	2.64	**0.008**	4.290915	12.07938	1.524246	8.45	**<0.001**
Chalcides sepsoides	6	0.77463	1.047336	0.572931	−5.08	**<0.001**	2.048894	4.567199	0.919155	5.37	**<0.001**
Chalcides ocellatus	6	1.03163	1.485335	0.716512	0.72	0.608	1.977479	4.591354	0.851693	4.86	**<0.001**
Myriopholis macrorhyncha	5	1.066423	2.030861	0.559988	0.5	0.618	2.745601	5.62657	1.339773	7.04	**<0.001**
Xerotyphlops syriacus	6	1.183765	1.687588	0.830357	2.85	**0.004**	2.784031	5.58397	1.38805	8.83	**<0.001**
Eryx jaculus	5	1.04656	1.48258	0.738772	0.53	0.513	3.717699	7.691263	1.797011	9.03	**<0.001**
Eirenis rothii	6	1.277557	2.175656	0.750189	2.76	**0.006**	3.020704	4.688206	1.9463	>10	**<0.001**
Eirenis decemlineatus	5	1.064761	1.474325	0.768973	0.97	0.335	3.013014	3.627055	2.502927	>10	**<0.001**
Lytorhynchus diadema	5	0.918145	1.79248	0.470293	−0.64	0.523	2.350434	7.268864	0.760028	3.79	**<0.001**
