# Supplementary material for: An Overlooked Habitat‐Dependent Link Between Metabolism and Water Loss in Reptiles
Source: Integr Zool. 2025 Jul 21;21(3):709–19. doi: 10.1111/1749-4877.13016 (PMC13164836; doi:10.1111/1749-4877.13016)
Supplement: Supplementary file 6 — Supporting Table 4 ValidaƟ on in two species of our CO2‐based results (showing higher RMR in dry air) against an O2‐based measure of RMR (using an Oxzilla O2 analyzer). [file INZ2-21-709-s005.pdf]

Table S4: Validation in two species of our CO2-based results (showing higher RMR in dry air) against an O2-based measure of RMR (using an Oxzilla O2 analyzer).

			RMR, humid air WVP=1		RMR, dry air WVP=3			
Species	ID	Mass	RMR_humid_O2	RMR_humid_CO2	RMR_dry_O2	RMR_dry_CO2	Block	Treatment_order
Eirenis rothii	1	2.6	34.46286	38.49072	44.53589	43.86899	A	dry-humid-humid-dry
Eirenis rothii	2	2.7	33.05426	25.76558	44.24380	34.83642	A	dry-humid-humid-dry
Eirenis rothii	3	2.7	25.65538	24.71372	34.45575	32.20858	A	dry-humid-humid-dry
Eirenis rothii	4	3.6	25.19453	25.32141	29.41155	31.15447	B	humid-dry-dry-humid
Eirenis rothii	5	2.1	35.61134	25.37775	34.47174	45.04578	B	humid-dry-dry-humid
Eirenis rothii	6	2.4	30.69936	28.46739	48.78441	36.34600	B	humid-dry-dry-humid
**mean**		**2.68**	**30.77962**	**28.02276**	**39.31719**	**37.24337**		
SD			4.461913028	5.293654668	7.567938613	5.895742046		
Xerotyphlops syriacus	1	2.5	30.83452	29.22913	32.99315	42.27617	B	humid-dry-dry-humid
Xerotyphlops syriacus	2	1.8	27.30133	26.45030	39.00980	31.99979	B	humid-dry-dry-humid
Xerotyphlops syriacus	3	1.5	31.74380	38.13222	35.93544	35.34965	B	humid-dry-dry-humid
Xerotyphlops syriacus	4	1.8	42.26709	27.69788	42.69902	39.05790	A	dry-humid-humid-dry
Xerotyphlops syriacus	5	1.9	28.08471	33.73948	35.54195	36.22567	A	dry-humid-humid-dry
Xerotyphlops syriacus	6	2.1	31.85806	25.35728	33.80236	27.17353	A	dry-humid-humid-dry
**mean**		**1.93**	**32.01492**	**30.10105**	**36.66362**	**35.34712**		
SD			5.371460927	4.901116305	3.616024307	5.302182848		
