# Supplementary material for: An Overlooked Habitat‐Dependent Link Between Metabolism and Water Loss in Reptiles
Source: Integr Zool. 2025 Jul 21;21(3):709–19. doi: 10.1111/1749-4877.13016 (PMC13164836; doi:10.1111/1749-4877.13016)
Supplement: Supplementary file 7 — Supporting Table 5: Multi‐species results of the PGLMMs for RMR and EWL. [file INZ2-21-709-s006.pdf]

Table S5: Multi-species results of the PGLMMs for RMR and EWL (R output, humid-mesic shown as intercept but all pairwise differences were examined and reported in the paper). "Species" is the random effect of species identity whereas "Species__" is the effect of their phylogenetic relationships based on the Zheng & Weins (2016) tree.

```
a) species means model: log10(RMR) ~ log10(Mass) * Treatment * Habitat
Random effects:
            Variance Std.Dev
1|Species   0.019110 0.13824
1|Species__ 0.002559 0.05059
residual    0.001689 0.04110
Fixed effects:
                                    Value Std.Error  Zscore    Pvalue
(Intercept)                      1.751061  0.090311 19.3892 < 2.2e-16 ***
log10(Mass)                      0.805757  0.086237  9.3435 < 2.2e-16 ***
Treatmentdry                     0.097895  0.032573  3.0054  0.002652 **
Habitatxeric                     0.086591  0.116400  0.7439  0.456930
log10(Mass):Treatmentdry        -0.054365  0.032379 -1.6790  0.093146 .
log10(Mass):Habitatxeric        -0.021033  0.138391 -0.1520  0.879199
Treatmentdry:Habitatxeric       -0.179252  0.043715 -4.1005 4.123e-05 ***
log10(Mass):Treatmentdry:Habitatxeric  0.125779  0.052678  2.3877  0.016955 *
R^2 = 0.9704197 (maximum likelihood)
```

```
b) all measurements model: log10(RMR) ~ log10(Mass) * Treatment * Habitat (+ random factors)
Random effects:
             Variance   Std.Dev
1|Species   7.570e-07 0.0008700
1|Species__ 3.789e-02 0.1946443
1|ID        5.710e-05 0.0075564
1|Order     1.356e-06 0.0011644
1|Block     2.018e-08 0.0001420
1|Replicate 1.824e-08 0.0001351
residual    2.399e-02 0.1548783

Fixed effects:
                                    Value Std.Error   Zscore  Pvalue
(Intercept)                     -1.286685  0.141799  -9.0740 < 2e-16 ***
log10(Mass)                      0.976226  0.066386  14.7052 < 2e-16 ***
Treatmentdry                     0.088555  0.034978   2.5317 0.01135 *
Habitatxeric                     0.032166  0.109890   0.2927 0.76974
Treatmentdry:log10(Mass)        -0.037718  0.036639  -1.0294 0.30327
log10(Mass):Habitatxeric         0.155720  0.101557   1.5333 0.12519
Treatmentdry:Habitatxeric       -0.141390  0.047271  -2.9910 0.00278 **
Treatmentdry:log10(Mass):Habitatxeric  0.063752  0.058695   1.0862 0.27741
R^2 = 0.9110698 (maximum likelihood)
```

c) species means model: log10(EWL) ~ log10(Mass) * Treatment * Habitat (+ random factors)

```
Random effects:
            Variance Std.Dev
1|Species   0.001857 0.04309
1|Species__ 0.007184 0.08476
residual    0.009105 0.09542
Fixed effects:
                                          Value Std.Error  Zscore    Pvalue
(Intercept)                           -0.340462  0.088204 -3.8599 0.0001134 ***
log10(Mass)                            0.773910  0.073655 10.5072 < 2.2e-16 ***
Treatmentdry                           0.583717  0.075626  7.7185 1.177e-14 ***
Habitatxeric                          -0.229093  0.102227 -2.2410 0.0250242 *
log10(Mass):Treatmentdry              -0.082619  0.075175 -1.0990 0.2717579
log10(Mass):Habitatxeric              -0.174141  0.117235 -1.4854 0.1374381
Treatmentdry:Habitatxeric             -0.213173  0.101495 -2.1003 0.0356991 *
log10(Mass):Treatmentdry:Habitatxeric  0.160037  0.122305  1.3085 0.1907018
R^2 = 0.9551707 (maximum likelihood)
```

d) all measurements model: log10(EWL) ~ log10(Mass) * Treatment * Habitat (+ random factors)

```
Random effects:
             Variance   Std.Dev
1|Species   1.991e-02 0.1411191
1|Species__ 6.096e-03 0.0780758
1|ID        1.807e-08 0.0001344
1|Order     6.755e-07 0.0008219
1|Block     1.634e-07 0.0004042
1|Replicate 1.008e-04 0.0100380
residual    4.713e-02 0.2171029
Fixed effects:
                                          Value Std.Error   Zscore    Pvalue
(Intercept)                           -0.342050  0.101798 -3.3601 0.0007792 ***
log10(Mass)                            0.673295  0.085024   7.9189 2.396e-15 ***
Treatmentdry                           0.513223  0.055830   9.1927 < 2.2e-16 ***
Habitatxeric                          -0.306506  0.123145  -2.4890   0.01281 *
Treatmentdry:log10(Mass)              -0.045598  0.058206  -0.7834   0.43340
log10(Mass):Habitatxeric               0.220104  0.125640   1.7519   0.07980 .
Treatmentdry:Habitatxeric             -0.146347  0.071440  -2.0485   0.04051 *
Treatmentdry:log10(Mass):Habitatxeric  0.059058  0.086715   0.6811   0.49584
R^2 = 0.8606874 (maximum likelihood)
```
